# Sugar-sweetened beverage taxation, oral health policy framing, and dental caries data availability: a global policy and evidence mapping study

**DOI:** 10.3389/froh.2026.1940529

**Published:** 2026-10-19

**Authors:** A. Vukovic, A. Arghittu, D. Lamloum, M. Dettori, C. Bertilsson, K. Gambetta-Tessini, P. Castiglia, P. Lingström, R. A. Giacaman, G. Campus

**Affiliations:** 1 Clinic for Paediatric and Preventive Dentistry, School of Dental Medicine, University of Belgrade, Belgrade, Serbia; 2 Department of Medicine, Surgery and Pharmacy, University of Sassari, Sassari, Italy; 3 Department of Restorative, Paediatric and Preventive Dentistry, University of Bern, Bern, Switzerland; 4 Graduate School for Health Sciences, University of Bern, Bern, Switzerland; 5 Heidelberg Institute of Global Health, Section for Oral Health, Heidelberg University, Heidelberg, Germany; 6 Department of Cariology, Institute of Odontology, Sahlgrenska Academy, University of Gothenburg, Gothenburg, Sweden; 7 Cariology Unit, Department of Oral Rehabilitation, Faculty of Dentistry, University of Talca, Talca, Chile; 8 Department of Odontostomatological and Maxillofacial Sciences, Sapienza Università di Roma, Rome, Italy

**Keywords:** dental caries prevention, oral health, prevention, public health policy, sugar-sweetened beverage tax

## Abstract

**Background:**

Sugar-sweetened beverage taxation (SSBT) is an upstream public health measure intended to reduce sugar intake and prevent diet-related non-communicable diseases. Although sugar consumption is strongly associated with dental caries, it remains unclear how often oral health is documented within SSBT policy frameworks and whether jurisdictions have sufficient epidemiological data to evaluate oral-health outcomes. This study assessed the availability of dental caries data among 12-year-olds in jurisdictions with SSBTs and examined whether oral health and/or dental caries were documented in policy rationales.

**Methods:**

This global evidence and policy mapping study identified sovereign states, territories, and jurisdictions with independently implemented SSBT-related fiscal policies. The availability of Decayed, Missing, and Filled Teeth (DMFT) data among 12-year-olds before and after SSBT implementation was assessed. SSBT laws and related implementation documents were reviewed for explicit or secondary references to oral health and dental caries. Jurisdictions were classified by World Health Organization region and income category, and per capita Gross National Income (pGNI) was used to assess economic status. Associations were analysed using Fisher's exact and Kruskal–Wallis tests.

**Results:**

SSBTs were identified in 129 countries, territories, or jurisdictions, of which 117 met the inclusion criteria. None of the reviewed SSBT legal texts explicitly mentioned oral health or dental caries as a legal objective. In related implementation documents, 22 jurisdictions (18.80%) included oral health or dental caries as explicit objectives and five (4.27%) as secondary benefits. Only 38 jurisdictions (32.48%) had DMFT data available both before and after SSBT implementation. The absence of documented oral-health references was significantly associated with lower median pGNI (*p* < 0.01), although this finding should be interpreted cautiously because of missing pGNI values and the small secondary-reference group. Explicit oral-health objectives were more frequent in jurisdictions within the WHO Western Pacific Region (*p* < 0.01).

**Conclusion:**

Oral-health references are absent from SSBT legal texts and inconsistently represented in related policy documents, although this does not demonstrate that oral health was absent from policy deliberations. Moreover, only a minority of jurisdictions have DMFT data available both before and after SSBT implementation, limiting the capacity to evaluate the oral-health outcomes of taxation policies.

## 1 Introduction

Sugar-sweetened beverages (SSBs) are one of the primary sources of free sugars in today's diet and the consumption is particularly widespread among children and adolescents (1). Sugar-sweetened beverage tax (SSBT) represents an effective upstream measure to reduce sugar-sweetened beverage (SSB) consumption and, consequently, control the development of diet-related chronic non-communicable diseases (NCDs) such as overweight, obesity, diabetes mellitus type 2, and dental caries (2, 3). SSBT has been introduced in many countries around the world since the middle of the 20th century (4, 5). However, certain policies, particularly those implemented prior to the 2000s, functioned primarily as general excise or consumption taxes rather than targeted public health interventions designed around sugar content (6). Recent SSBTs are health-oriented and have a dual objective: to discourage the consumption of sugars aimed at shifting consumers' dietary habits through an increase in prices and/or to generate tax revenues to be reinvested in public health care programs and NCDs prevention (7, 8). In high-income countries (HICs), these taxes are part of integrated strategies to combat NCDs, while in upper and lower middle- and low-income economies (UMICs, LMICs and LICs) they may represent an important source of financing for health systems under pressure (9, 10).

Robust econometric and risk assessment modeling studies provided scientific evidence on SSBTs' efficacy and cost-effectiveness in reducing NCDs such as obesity and overweight (6, 11, 12). Also, econometric modeling studies and global reviews confirmed that implementing fiscal policies on SSBs served as an effective oral health strategy, mitigating the burden of dental caries and minimizing dental treatment expenditures across diverse healthcare systems (13–18). However, despite being considerate as a preventable disease, dental caries is still one of the most prevalent chronic NCDs (19). Unfortunately, dental caries and/or oral health in general are often neglected in most national health systems (20). In order to enable SSBT introduction, monitoring and evaluation of the SSBT outcomes, it is necessary to assess the availability of epidemiological dental caries experience data.

Therefore, this study aimed to conduct a global evidence and policy mapping analysis among countries and jurisdictions with SSBTs. Specifically, we aimed: first, to assess the availability of epidemiological dental caries data, based on DMFT in 12-year-old children, before and after SSBT implementation; and second, to determine whether dental caries and/or oral health were explicitly or secondarily incorporated into SSBT laws and related implementation documents. The study does not aim to estimate the causal effect of SSBTs on dental caries, but rather to evaluate whether current data availability and policy framing allow such evaluation to be conducted.

## 2 Methods

### 2.1 Study design

This study was designed as a global evidence and policy mapping study. It combined: (i) identification of countries with documented SSBTs; (ii) a systematic search for epidemiological DMFT data in 12-year-old children before and after SSBT implementation; and (iii) a structured documentary analysis of SSBT laws and related implementation documents to identify whether dental caries and/or oral health were included as policy objectives or expected benefits.

### 2.2 Information sources and search strategy

The survey was planned and conducted in three phases (Table 1, and Figure 1):

1. The first phase was focused on the identification of countries that adopted SSBT.
2. The second phase was based on a systematic literature search to identify the availability of dental caries experience epidemiological data in countries before SSBT and at least three years after the SSBT implementation.
3. The third phase was conducted to identify the presence of “oral health and/or dental caries” terminology in any place in the SSBT rationale in the included countries in SSBT laws and implementation documents.

**Figure 1:**
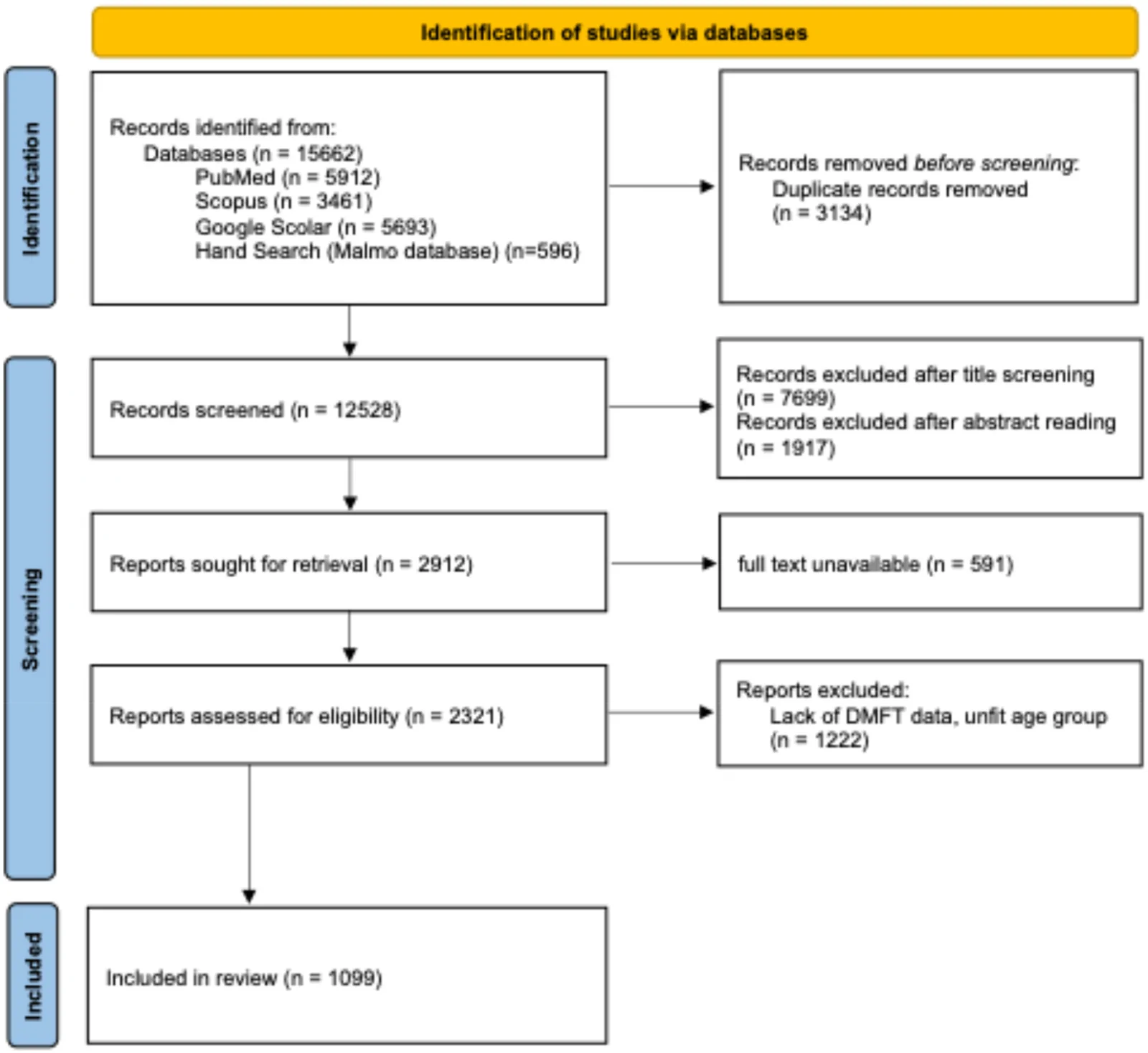
PRISMA-flowchart of the review process.

**Table 1:** Systematic scientific literature search strategy.

Database	Search string
Pubmed	(“country” [tw]) AND (“Dental Caries”[MeSH] OR “DMF Index”[MeSH] OR “Oral Health”[MeSH] OR “dental caries”[tw] OR “oral health”[tw]) AND (Child [MeSH] OR child* [All Fields] OR Adolescent [MeSH] OR adolescent [tw] OR 12?year*[All Fields])
Scopus	TITLE-ABS-KEY({country}) AND TITLE-ABS-KEY({dental caries} OR {oral health}) AND TITLE-ABS-KEY(child*[All Fields] OR {adolescent})
Google Scholar	“country” AND (“dental caries” OR “oral health” OR “DMF Index”) AND (child* OR adolescent or 12-year*)

Jurisdictions that had adopted SSBTs were identified via the Obesity Evidence Hub (5) and the World Bank Global SSB Tax Database (4). For this study, the unit of analysis was a jurisdiction with a documented SSBT policy framework and sufficient jurisdiction-level information for classification. This definition included sovereign states as well as territories or other jurisdictions with independent fiscal or public-health authority to implement an SSBT-related policy. The term “jurisdiction” is therefore used below as an umbrella term unless a distinction between sovereign states and territories is specifically required. Policies were retained regardless of the period in which they were established or their original legislative intent.

A search strategy regarding DMFT data availability was formulated as described in Table 1 with both Medical Subject Headings (MeSH) and non-MeSH terms, conducting a comprehensive search of three electronic databases: PubMed, Scopus, and Google Scholar. For Google Scholar searches, results were screened in order of relevance up to a predefined maximum number of records per country, or until no additional eligible epidemiological DMFT data sources were identified. The number of Google Scholar records screened and the stopping rule should be reported to improve reproducibility. For each country, the first 100 Google Scholar results were screened unless no new relevant records appeared after several consecutive pages.

A hand search was also performed, exploiting backward citation, snowball sampling, the WHO-CAPP Malmö Database (21), and data derived from doctoral and master theses in native national languages. If missing data were present, the corresponding authors were contacted directly, if feasible. Following the conclusion of the data extraction process, a random selection of 10% of the papers was conducted by one author (AV) to verify the accuracy of the data extraction procedure.

A thorough examination of the pertinent laws and implementation documents relating to the SSBT has been conducted to ascertain the presence of references to oral health and/or dental caries (OHDC). Subsequently, a comprehensive search was conducted of the implementation documents as governmental reports, background policy analyses and strategic health plans using the WHO Global Database on the Implementation of Food and Nutrition Action (GIFNA), Ministries of Health and government websites for each country, as well as the Country Cooperation Strategy (CCS), the WHO Global Health Observatory (GHO), the Organisation for Economic Co-operation and Development (OECD) web pages and documents. (details on analysed legislative documents is reported in the Supplementary Appendix Table 1).

### 2.3 Eligibility criteria

Inclusion and exclusion criteria for the selection of articles and policy documents in all phases were established before the search. Analyses were conducted at the level of the eligible SSBT jurisdiction. Sovereign states, territories, and other jurisdictions were eligible when they had an independently documented SSBT-related fiscal policy and sufficient jurisdiction-level information for the study variables. Supra-jurisdictional regional aggregates were not treated as units of analysis.

#### 2.3.1 Systematic scientific literature search

*The inclusion criteria:* observational epidemiological and/or ecological surveys including samples of 12-year-olds without a date/year filter. The date/year filter was excluded considering that some SSBTs were introduced in the middle of the 20th century and some recently. The 12-year-old age group was selected based on the WHO's recommendation to use this age group as a standard indicator for international epidemiological comparisons in oral health surveys. At this age, most children have a complete permanent dentition, and they are the oldest group still attending primary schools in most countries (22). Additionally, children of this age demonstrate greater dietary independence from their parents and are more likely to purchase and consume SSBs independently (23). This makes them a particularly relevant population for assessing the impact of taxation policies on beverage consumption patterns and subsequent oral health outcomes.

*The exclusion criteria* applied to papers if the full text could not be downloaded, the downloaded document was unreadable, epidemiological caries data (DMFT) were not used or could not be calculated, or the surveys were conducted in countries where SSBT was not implemented.

#### 2.3.2 Oral health information availability and documentary analysis

*The inclusion criteria* applied to official or institutional documents produced as part of the SSBT policy process. The data were gathered until May of 2025. To enhance consistency and reduce interpretive bias, two researchers (AA and PC) independently reviewed and coded a subset of documents. Discrepancies were resolved through consensus.

Eligible SSBT-related laws and implementation documents were retained regardless of whether they mentioned oral health. Documents were coded as “oral health not addressed” when no relevant dental caries or oral-health terminology was identified. Documents were excluded only if they were not related to SSBT policy, could not be retrieved in full text, or could not be interpreted after translation and review.

Moreover, isolated mentions of these terms in SSBT-unrelated documents were not considered sufficient for inclusion.

### 2.4 Data sources and independent variables

The following independent variables were recorded: year of data collection in each included study and respective dental caries epidemiological data value (DMFT) and year when SSBT was introduced. As SSBTs vary substantially in design and original policy intent, taxes were classified according to their apparent policy orientation when information was available: general beverage excise/consumption tax, health-oriented SSBT, sugar-content-based SSBT, or mixed fiscal-health policy. This classification was used descriptively to contextualize policy heterogeneity. Historical taxes introduced before the 2000s were interpreted cautiously, as several of these measures were not originally designed as health-oriented interventions. Moreover, the World Bank database was consulted to collect the *per capita* Gross National Income (pGNI) of the countries in the year of SSBT introduction. All countries were categorized according to income level using World Bank classification in United States Dollars (USD): LICs ≤1,145, LMICs 1,146–4,515, UMICs 4,516–14,005, HICs ≥14,006 (24). For analyses involving pGNI, the seven countries/jurisdictions without an observed pGNI value were excluded and a complete-case analysis was performed; no regional-mean imputation was used in the revised analysis.

Countries were also categorized according to the World Health Organization (WHO) database: WHO African Region (AFRO), WHO Region of the Americas (AMRO/PAHO), WHO South-East Asia Region (SEARO), WHO European Region (EURO), WHO Eastern Mediterranean Region (EMRO), and WHO Western Pacific Region (WPRO) (25).

### 2.5 Outcome variables

A coding framework was developed before data extraction. A reference was coded as “explicit” when dental caries or oral health was directly stated as an objective, expected outcome, or justification of the SSBT policy; for example, wording that identified reduction of dental disease or oral-health problems as an intended policy benefit. A reference was coded as “secondary” when oral health was documented as an indirect or collateral benefit of reducing sugar consumption but was not presented as a primary objective or justification. A document was coded as “oral health not addressed” when no reference to dental caries, oral health, oral hygiene, dental disease, or related terminology was identified. This category denotes absence of a documented reference in the material reviewed and does not imply that oral health was absent from policy deliberations. Where more than one eligible document was available for a jurisdiction, legal texts and related implementation/policy documents were reviewed separately; the jurisdiction-level policy-document classification reflected the highest level of documented oral-health reference identified (explicit, then secondary, then not addressed), while the legal-text finding was reported separately. A subset of documents was independently coded by two reviewers, and disagreements were resolved by discussion and consensus. Formal inter-rater agreement statistics were not calculated; therefore, the coding should be interpreted as structured consensus coding.

- DMFT data availability was classified by comparing the year of DMFT data collection with the year of SSBT implementation. Countries were categorized as having: (i) DMFT data only before SSBT implementation; (ii) DMFT data only after SSBT implementation; (iii) DMFT data both before and after SSBT implementation; or (iv) no eligible DMFT data identified. The presence of DMFT data before and after SSBT implementation was interpreted as an indicator of potential evaluative capacity, not as evidence that the available data were directly comparable or sufficient for causal policy evaluation.
- The countries were then categorized as having any mention of dental caries and/or oral health in the text of the SSBT law and other policy documents.
- The countries were coded as having: (a) an explicit oral health reference if the terms “dental caries and/or oral health” were directly stated as a policy objective or expected outcome of SSBT; (b) a secondary oral health reference if the terms were mentioned indirectly as a potential collateral benefit of reducing sugar consumption, or (c) oral health was not addressed at all.
- The countries were coded as those with health and economic reasons for introducing SSBT and those with only health reasons.

### 2.6 Data analysis

All data were inserted in *Microsoft* Excel sheet and analysed using SPSS® version 26 (SPSS Inc, Chicago, IL).

Descriptive statistics were used to summarize the distribution of SSBT countries by WHO region, World Bank income category, DMFT data availability, oral-health reference category, and SSBT policy objective articulation. Fisher's exact test was used to examine associations between categorical variables, including WHO region and oral-health reference category, WHO region and policy objective articulation, and income category and policy objective articulation. Because pGNI was not normally distributed, Kruskal–Wallis tests were used to compare pGNI distributions across oral-health reference categories. Results are reported as counts, percentages, medians, ranges, and mean ranks where appropriate. A two-sided *p* value <0.05 was considered statistically significant. To explore whether oral-health policy framing differed across historical periods, SSBT implementation years were grouped *a priori* into three eras: before 2000, 2000–2014, and 2015–2023. The distribution of oral-health reference categories (explicit, secondary, and oral health not addressed) was compared across these periods using the Fisher-Freeman-Halton exact test for the 3 × 3 contingency table. This temporal analysis was interpreted as an association with the era of SSBT introduction rather than as evidence that later global health initiatives causally changed policy framing. For the pGNI comparison, only units with observed pGNI values were included (complete-case *n* = 110). Because pGNI was not normally distributed, the Kruskal–Wallis test was used to compare pGNI distributions across the three oral-health reference categories.

## 3 Results

### 3.1 Study selection

The flowchart of the included papers and selection process is presented in Figure 1.

A total of 129 countries, territories, or jurisdictions with documented SSBTs were initially identified. After applying the eligibility criteria, 117 countries were included in the final analysis. Exclusions were due to insufficient country-level information, duplicate or overlapping policy entries, or lack of an eligible SSBT policy framework.

SSBTs were introduced from 1965 to 2023. Countries with SSBTs were mostly from WHO AFRO (*n* = 35, 29.91%), followed by WHO AMRO/PAHO (*n* = 23, 19.66%), WHO EURO (*n* = 21, 17.95%) and WHO WPRO (*n* = 21, 17.95%). The least frequent were countries from WHO SEARO (*n* = 7, 5.98%) and WHO EMRO (*n* = 10, 8.55%) (the characteristics are reported in the Supplementary Appendix Table 1). Only two countries (1.71%) had dental caries epidemiological data available only after SSBT introduction, 68 countries (58.12%) had oral health data only before SSBT implementation, and 9 countries had no data at all (7.69%). Country-level DMFT data availability in relation to SSBT implementation—before, after, both, or neither—is also reported in Supplementary Appendix Table 1, together with the corresponding DMFT values and survey years. Among the 117 countries and jurisdictions, eligible age-12 DMFT data were available only before SSBT implementation for 73 (62.4%), both before and after implementation for 25 (21.4%), only after implementation for 7 (6.0%), and neither before nor after implementation for 12 (10.3%). Overall, pre-SSBT DMFT estimates were more frequently available than post-SSBT estimates (83.8% vs. 27.4%), indicating substantial variation in the temporal coverage of oral-health surveillance across countries. These availability categories describe the presence and timing of DMFT data and should not be interpreted as evidence of a causal effect of SSBT implementation on dental caries.

### 3.2 Documentary analysis of oral health positioning within SSBT policies

None of the SSBT legal texts reviewed included dental caries or oral health as an explicit legal objective. However, in related implementation or policy documents, 22 countries included explicit oral-health or dental-caries references, and five included secondary references (Figure 2); 22 (18.80%) countries reported an explicit dental caries and/or oral health reference in the other policy documents and five countries (4.27%) addressed dental caries and/or oral health as a secondary objective (the characteristics are reported in the Supplementary Appendix Table 2).

**Figure 2:**
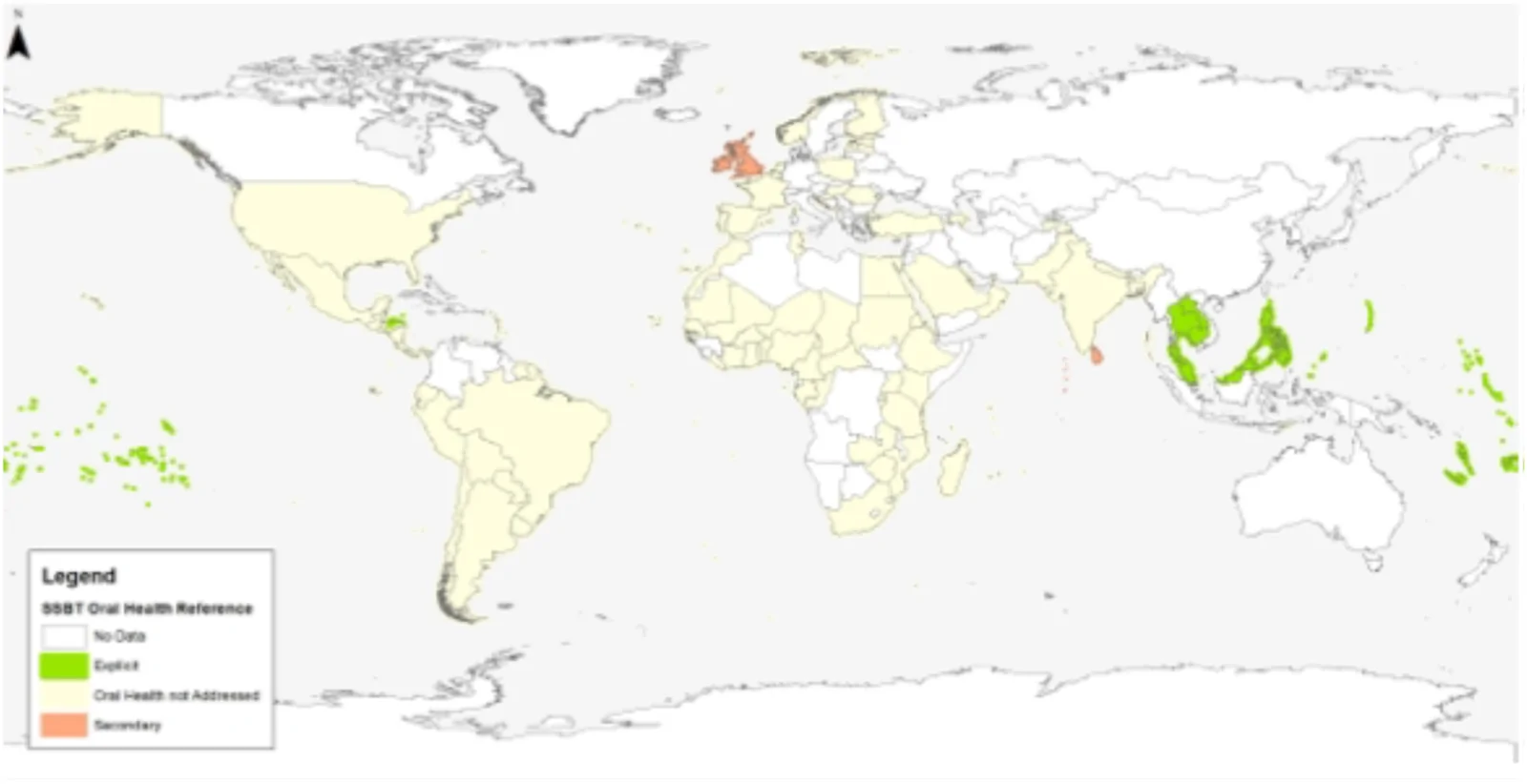
Map of SSBT countries SSBT countries based on whether their SSBT policy documents mention dental caries and/or oral health.

Almost half of all countries with SSBT from WPRO WHO region had explicit oral health reference within SSB taxation showing statistically significant difference compared to countries with SSBT from other WHO regions (Table 2).

**Table 2:** Distribution of countries by WHO region and SSBT oral health reference.

WHO region	SSBT oral health reference			Total count (%)
	Explicit count (%)	Secondary count (%)	Oral health not addressed count (%)	
AFRO	0 (0.00)	0 (0.00)	35 (100.00)	35 (100.00)
AMRO/PAHO	1 (4.35)	0 (0.00)	22 (95.65)	23 (100.00)
SEARO	1 (14.29)	2 (28.57)	4 (57.14)	7 (100.00)
EURO	0 (0.00)	3 (14.29)	18 (85.71)	21 (100.00)
EMRO	0 (0.00)	0 (0.00)	10 (100.00)	10 (100.00)
WPRO	20 (95.24)	0 (0.00)	1 (4.76)	21 (100.00)
Total	22 (18.80)	5 (4.27)	90 (76.93)	117 (100.00)

Fisher's exact test = 90.59, *p* < 0.01.

Further analysis showed that health reasons for SSBT introduction were the most frequent in AFRO, AMRO/PAHO, and EMRO WHO region (100% countries with SSBT) (Table 3). However, WPRO region revealed that most of all countries with SSBT (80.95%) stated both health and economic reasons vs. only health reasons (*p* < 0.01) (Table 3).

**Table 3:** Distribution of countries by WHO region and SSBT objective articulation.

WHO region	SSBT policy objective articulation		Total count (%)
	Health and economic reasons count (%)	Health reasons count (%)	
AFRO	0 (0.00)	35 (100.00)	35 (100.00)
AMRO/PAHO	0 (0.00)	23 (100.00)	23 (100.00)
SEARO	1 (14.28)	6 (85.71)	7 (100.00)
EURO	2 (9.52)	19 (90.48	21 (100.00)
EMRO	0 (0.00)	10 (100.00)	10 (100.00)
WPRO	17 (80.95)	4 (19.05)	21 (100.00)
Total	20 (17.09)	97 (82.91)	117 (100.00)

Fisher's exact test = 58.71, *p* < 0.01.

Results revealed that both health and economic reasons for SSBT introduction were significantly (*p* < 0.05) more frequent in UMICs (36.00%) compared to other income categories as presented in Table 4.

**Table 4:** Association between country income category and SSBT objective articulation.

Country income category	SSBT policy objective articulation		Total count (%)
	Health and economic reasons count (%)	Health reasons count (%)	
LIC	2 (8.00)	23 (92.0)	25 (100.00)
LMIC	4 (9.80)	37 (90.20)	41 (100.00)
UMIC	9 (36.00)	16 (64.00)	25 (100.00)
HIC	5 (19.20)	21 (80.80)	26 (100.00)
Total	20 (17.10)	97 (82.90)	117 (100.00)

Fisher's exact test = 8.37, *p* < 0.05.

In the complete-case analysis, 110 countries/jurisdictions with observed pGNI values were included and seven units with unavailable observed pGNI were excluded. Median pGNI was USD 3,970 in the explicit-reference group (*n* = 18), USD 42,130 in the secondary-reference group (*n* = 5), and USD 2,150 in the oral-health-not-addressed group (*n* = 87). pGNI distributions differed significantly across the three oral-health reference categories (Kruskal–Wallis H(2) = 7.68, *p* = 0.022) (Table 5). This finding should be interpreted cautiously because the secondary-reference group included only five units and pGNI values showed wide dispersion.

**Table 5:** Median of pGNI stratified by SSBT oral health reference.

Oral health reference	Count (*n*)	Median pGNI (USD)	Min-max	Mean rank
Explicit	18	3,970.00	370–35,550	61.03
Secondary	5	42,130.00	4,350–84,470	91.20
Oral Health not Addressed	87	2,150.00	110–1,17,160	52.30

Complete-case *n* = 110; Kruskal–Wallis H(2) = 7.68, *p* = 0.022.

### 3.3 Historical analysis of oral-health policy framing

SSBT implementation years were distributed across the three prespecified historical periods as follows: 16 countries/jurisdictions before 2000, 45 during 2000–2014, and 56 during 2015–2023. Explicit oral-health references were identified in 4/16 (25.0%), 10/45 (22.2%), and 8/56 (14.3%) units, respectively. Secondary references were absent in the first two periods and occurred in 5/56 (8.9%) units during 2015–2023, while oral health was not addressed in 12/16 (75.0%), 35/45 (77.8%), and 43/56 (76.8%), respectively. The distribution of oral-health reference categories did not differ significantly across historical periods (Fisher-Freeman-Halton exact test, *p* = 0.201) (Table 6). Thus, the data did not demonstrate a clear temporal shift toward more frequent explicit oral-health framing in more recently introduced SSBTs.

**Table 6:** Distribution of SSBT oral health reference by historical period of SSBT introduction.

Historical period	Explicit *n* (%)	Secondary *n* (%)	Oral health not addressed *n* (%)	Total *n* (%)
Before 2,000	4 (25.0)	0 (0.0)	12 (75.0)	16 (100.0)
2000–2014	10 (22.2)	0 (0.0)	35 (77.8)	45 (100.0)
2015–2023	8 (14.3)	5 (8.9)	43 (76.8)	56 (100.0)
Total	22 (18.8)	5 (4.3)	90 (76.9)	117 (100.0)

Fisher-Freeman-Halton exact test, *p* = 0.201.

## 4 Discussion

This global evidence and policy mapping study examined whether countries with SSBTs have the epidemiological caries data and policy framing needed to evaluate oral-health outcomes. Three main findings emerged. First, only a minority had DMFT data for 12-year-olds both before and after SSBT implementation. Second, dental caries and oral health were absent from the legal texts of SSBTs reviewed. Third, oral health was mentioned in related policy or implementation documents in only a limited number of countries, with marked regional variation. By combining a structured review of policy documents with a systematic assessment of epidemiological data availability, this study sought to explore the alignment between scientific evidence linking sugar consumption to dental caries and the way such evidence is translated into fiscal public health policies. This dual focus addresses a critical gap in the literature. Although SSBTs are increasingly promoted as effective tools for tackling obesity, diabetes, and other NCDs, the impacts on oral health outcomes are rarely considered within the same policy framework (26). The historical analysis did not identify a statistically significant association between the era of SSBT introduction and oral-health reference category. Explicit oral-health references were not more frequent among policies introduced in 2015–2023 than among those introduced before 2000 or during 2000–2014; the five secondary references were confined to the most recent period. These findings do not support a simple progressive increase in documented oral-health framing over time. However, the implementation year of a tax is not necessarily the publication year of every policy or implementation document reviewed, and older fiscal measures may subsequently have been discussed within newer health-policy frameworks. The temporal findings should therefore be interpreted as descriptive evidence of policy-era heterogeneity, not as a causal assessment of the influence of global NCD or oral-health initiatives. Beverage taxes capturing SSBs long predate the current wave of health-motivated levies: the World Bank's Global SSB Tax Database records introduction dates of 1965 for Brazil, 1969 for Niue and 1976 for São Tomé and Príncipe (27, 28). These reflect when a taxing instrument took effect, not early health policy. Many are broad excise or import duties, and almost one-third of SSB excise taxes worldwide tax unsweetened bottled water at equal or higher rates. Brazil is illustrative: country-level analyses describe the IPI as a general manufactured-goods levy and conclude Brazil has no SSB-specific tax, its rate having fallen in 2016 and 2018 (29).

The findings of this global analysis revealed several consistent and concerning patterns. First, substantial gaps were identified in the availability of epidemiological caries data needed to evaluate the impact of SSBTs on dental caries outcomes. Only few countries had DMFT data available for 12-year-olds both before and after SSBT implementation. In contrast, more than half of the countries had dental caries data only prior to the introduction of SSBT, and little few had no identifiable data at all. The limited availability of post-SSBT dental caries data further undermines the ability to integrate oral health into policy evaluation. Without longitudinal epidemiological surveillance, even countries that recognize oral health as a potential benefit of SSBT lack the empirical basis to demonstrate impact (30, 31). This contrasts with obesity and diabetes outcomes, which are often monitored through established national surveillance systems (32). The resulting evidence asymmetry may reinforce the exclusion of oral health from future policy considerations, creating a self-perpetuating cycle of neglect.

Second, none of the 117 countries included in the analysis explicitly mentioned dental caries and/or oral health within the law text of their SSBT legislation. This absence was universal, regardless of WHO region or national income category. Second, when a broader range of policy-related documents was examined—such as governmental reports, background policy analyses, and strategic health plans—only 27 countries referenced dental caries and/or oral health in relation to SSBT. Among these, 22 countries articulated oral health as an explicit objective of sugar taxation, while five countries addressed oral health as a secondary or collateral benefit.

Third, notable regional and economic patterns emerged. Countries in the WHO Western Pacific Region (WPRO) were significantly more likely to include explicit oral-health references in SSBT-related policy documents and to frame SSBTs as serving both health and economic purposes. In the revised complete-case analysis, pGNI distributions also differed significantly across oral-health reference categories (Kruskal–Wallis H(2) = 7.68, *p* = 0.022), with the lowest median pGNI observed among units in which oral health was not addressed. This ecological association should not be interpreted as evidence that national income determines policy framing, particularly given the small secondary-reference group and the wide dispersion of pGNI values.

The near-total absence of documented oral-health references in the SSBT legal texts reviewed stands in contrast to the robust scientific evidence linking sugar consumption—particularly from SSBs—to dental caries (28, 30, 31). International health authorities, including the WHO, have emphasized the importance of reducing sugar intake for caries prevention (26). However, the documentary nature of the present analysis is important: failure to identify explicit terminology in legislation or related documents does not establish that oral health was absent from policy deliberations, where it may have been considered implicitly within broader objectives concerning sugar consumption, nutrition, or NCD prevention. The findings therefore concern documented policy framing rather than the full decision-making process.

This disconnect reflects a broader historical marginalization of oral health within public health systems (20). In many countries, oral health services are delivered separately from general health care, often financed through private expenditure or limited public coverage (33). As a result, oral diseases are frequently excluded from national NCD strategies, even though they share etiological pathways with conditions such as obesity and type 2 diabetes. SSBTs, which are often justified by the need to curb obesity-related health care costs or to generate revenue for health systems, thus appear to replicate this separation by focusing narrowly on metabolic outcomes.

At the same time, the regional differences observed in this study suggest that national health policy contexts shape how SSBTs are conceptualized and justified. The strong concentration of explicit oral-health references in the WPRO countries may partly reflect the inclusion of Pacific Island countries, where policy discussions around SSBs often explicitly connect sugar consumption with dental disease, obesity, diabetes, and health-system financing. This clustering should be interpreted as a regional policy pattern rather than as evidence of independent country-level effects. Here, SSBTs are not just considered economic or behavioural interventions, but also essential tools for reducing the burden of preventable diseases throughout one's lifetime. However, the results of the present study also revealed significantly lower pGNI in countries with oral health not addressed behind SSBT. This may be explained by the lack of capacity to develop comprehensive, multi-sectoral health policies that link fiscal measures to specific health outcomes. The complete-case pGNI analysis suggests an association between economic context and documented oral-health framing, but the pattern is not monotonic and should not be overinterpreted. The secondary-reference group had the highest median pGNI but contained only five units, while the explicit-reference group showed a substantially lower median. Differences in health-system organization, policy priorities, surveillance capacity, and the historical purpose of individual taxes may all contribute to the observed distribution. Accordingly, these cross-sectional country/jurisdiction-level data do not support causal claims about the effect of national income on whether oral health is incorporated into SSBT policy documents. The pGNI analysis was therefore revised as a complete-case analysis to avoid dependence on regional-mean imputation; seven units without observed pGNI values were excluded.

The study highlights a missed opportunity to leverage SSBTs as truly integrated public health interventions. By explicitly incorporating oral health objectives and indicators into SSBT frameworks, policymakers could enhance the cost-effectiveness and political appeal of these measures (34). Moreover, routine inclusion of dental caries surveillance alongside metabolic outcomes would generate a more comprehensive evidence base to inform future policy decisions.

From a global perspective, aligning SSBTs with the WHO Global Strategy on Oral Health (34) and the common risk factor approach to NCD prevention could help elevate oral health within broader health agendas. Such integration is particularly important for low- and middle-income countries, where preventive policies may offer the greatest returns on investment in the context of limited treatment capacity.

## 5 Conclusion

This global evidence and policy mapping study demonstrates a substantial gap between the scientific evidence linking sugar consumption to dental caries and the explicit documentation of oral health within SSBT policy frameworks. Dental caries and oral health were not identified as explicit objectives in the SSBT legal texts reviewed and were mentioned in related policy documents in only a minority of jurisdictions. This absence of documented references should not be interpreted as evidence that oral health was absent from policy deliberations. Moreover, only about one-third of jurisdictions had DMFT data for 12-year-olds both before and after SSBT implementation, limiting readiness to evaluate oral-health outcomes. Integrating oral-health objectives and surveillance indicators into SSBT frameworks would strengthen the accountability, comprehensiveness, and public-health value of these policies.

## Data Availability Statement

All data generated or analysed during this study are included in this article and its supplementary material files. Further enquiries can be directed to the corresponding author.
