# Supplementary material for: Sugar-sweetened beverage taxation, oral health policy framing, and dental caries data availability: a global policy and evidence mapping study
Source: Front Oral Health. 2026 Oct 19;7:1940529. doi: 10.3389/froh.2026.1940529 (PMC13728781; doi:10.3389/froh.2026.1940529)
Supplement: Supplementary file 1 [file Table1.docx]

**SUPPLEMENTARY MATERIAL**

**“Sugar-Sweetened Beverage Taxation, Oral Health Policy Framing, and Dental Caries Data Availability: A Global Policy and Evidence Mapping Study.”**

| **Contents** | | |
| --- | --- | --- |
|  |  | Page |
| Appendix Table 1. | Socio-economic characteristics of countries and jurisdictions implementing sugar-sweetened beverage taxes (SSBTs). | 2 |
| Appendix Table 2. | SSBT characteristics in countries implementing policy. | 5 |
| References |  | 12 |

Appendix Table 1. Socio-economic characteristics and pre- and post-SSBT dental-caries experience (DMFT at age 12) of countries/jurisdictions implementing sugar-sweetened beverage taxes (SSBTs).

|  | Country/jurisdiction  (Name) | Year of SSBT implementation | GNI *per capita,* USD  (*year of SSBT implementation*) | WHO Region | Pre-SSBT DMFT (age 12) | Pre-SSBT survey year | Post-SSBT DMFT (age 12) | Post-SSBT survey year |
| --- | --- | --- | --- | --- | --- | --- | --- | --- |
| 1. | American Samoa | 2001 | 8820 | WPRO | NA | — | NA | — |
| 2. | Argentina | 1996 | 7680 | AMRO / PAHO | **3.4** | 1987 | 3.0 | 2014-2015 |
| 3. | Azerbaijan | 2019 | 4510 | EURO | NA | — | NA | — |
| 4. | Bahrain | 2017 | 22360 | EMRO | **2.26** | 2011-2012 | NA | — |
| 5. | Bangladesh | 2012 | 950 | SEARO | **1.0** | 2000 | NA | — |
| 6. | Barbados | 2015 | 18010 | AMRO / PAHO | **0.86** | 2001 | NA | — |
| 7. | Belgium | 2009 | 47120 | EURO | **0.92** | 2001 | 0.8 | 2012-2014 |
| 8. | Belize | 2000 | 4480 | AMRO / PAHO | **0.65** | 1999 | NA | — |
| 9. | Benin | 2011 | 1030 | AFRO | **0.7** | 1994 | NA | — |
| 10. | Bermuda | 2018 | 117160 | AMRO / PAHO | **0.54** | 2009 | NA | — |
| 11. | Bolivia | 2016 | 3000 | AMRO / PAHO | **4.6** | 2015 | NA | — |
| 12. | Brazil | 1965 | 270 | AMRO / PAHO | NA | — | 1.9 | 2015 |
| 13. | Brunei Darussalam | 2017 | 29230 | WPRO | **0.9** | 2015-2016 | NA | — |
| 14. | Burkina Faso | 1995 | 250 | AFRO | **1.7** | 1993 | 0.7 | 1999 |
| 15. | Burundi | 2012 | 230 | AFRO | **1.0** | 1987-1988 | 1.41 | 2022-2023 |
| 16. | Cabo Verde | 2019 | 4420 | AFRO | **2.8** | 1989 | NA | — |
| 17. | Cambodia | 2003 | 370 | WPRO | **1.6** | 1990 | 3.5 | 2011 |
| 18. | Cameroon | 2019 | 1560 | AFRO | **0.28** | 2018 | NA | — |
| 19. | Central African Republic | 2019 | 500 | AFRO | **4.1** | 1986 | NA | — |
| 20. | Chad | 2019 | 920 | AFRO | NA | — | NA | — |
| 21. | Chile | 2014 | 14690 | AMRO / PAHO | **2.91** | 2011 | NA | — |
| 22. | Congo, Rep. | 2018 | 2070 | AFRO | NA | — | NA | — |
| 23. | Cook island | 2014 | 8820 | WPRO | **4.9** | 1978 | 1.7 | 2014-2018* |
| 24. | Costa Rica | 2001 | 3700 | AMRO / PAHO | **5.5** | 1992 | 2.5 | 2006 |
| 25. | Côte d'Ivoire | 2018 | 2010 | AFRO | **1.2-2.0** | 1996 | NA | — |
| 26. | Croatia | 1994 | 13580 | EURO | **2.6** | 1991 | 3.4 | 2015-2016 |
| 27. | Dominica | 2015 | 7510 | AMRO / PAHO | **1.2** | 2006 | NA | — |
| 28. | Ecuador | 2016 | 5750 | AMRO / PAHO | **1.61** | 2009-2010 | NA | — |
| 29. | Egypt | 2016 | 3200 | EMRO | **1.3** | 2016 | NA | — |
| 30. | El Salvador | 2010 | 2940 | AMRO / PAHO | **1.40-1.45** | 2008 | NA | — |
| 31. | Eritrea | 2001 | 320 | AFRO | NA | — | 2.5 | 2017 |
| 32. | Estonia | 2018 | 21600 | EURO | **2.0** | 2018 | NA | — |
| 33. | Ethiopia | 2003 | 110 | AFRO | **1.0** | 1993 | NA | — |
| 34. | Fiji | 1986 | 1740 | WPRO | **2.8** | 1986 | 1.54 | 1998 |
| 35. | Finland | 2001 | 25840 | EURO | **1.1** | 1997 | 0.8-0.9 | 2021 |
| 36. | France | 2012 | 43500 | EURO | **1.2** | 2006 | NA | — |
| 37. | French Polynesia | 2004 | 19380 | WPRO | **3.2** | 1994 | NA | — |
| 38. | Gabon | 2013 | 8870 | AFRO | **4.9** | NR (pub. 2010) | NA | — |
| 39. | Gambia | 2010 | 830 | AFRO | **2.27** | 2003 | NA | — |
| 40. | Ghana | 2014 | 1830 | AFRO | **0.4** | 1997-1998 | NA | — |
| 41. | Grenada | 2023 | 10110 | AMRO / PAHO | **5.2** | 2000 | NA | — |
| 42. | Guatemala | 2002 | 1590 | AMRO / PAHO | **2.4-8.1** | 1987-1988 | NA | — |
| 43. | Guinea-Bissau | 2022 | 950 | AFRO | **0.5** | 1986 | NA | — |
| 44. | Honduras | 2018 | 2280 | AMRO / PAHO | **3.7** | 2008 | NA | — |
| 45. | Hungary | 2011 | 13260 | EURO | **3.8** | 1996 | 2.32 | 2016-2017 |
| 46. | India | 2017 | 1790 | SEARO | **1.6** | 2012 | NA | — |
| 47. | Ireland | 2018 | 59120 | EURO | **1.1** | 2002 | NA | — |
| 48. | Isle of Man | 2019 | 84470 | EURO | NA | — | NA | — |
| 49. | Kenya | 2015 | 1320 | AFRO | **0.4** | 2015 | NA | — |
| 50. | Kiribati | 2014 | 3260 | WPRO | **1.0** | 1994 | NA | — |
| 51. | Lao PDR | 2005 | 450 | WPRO | **1.9** | 1991 | 1.59 | 2019 |
| 52. | Latvia | 2000 | 3240 | EURO | **5.7** | 1998 | 2.2 | 2021 |
| 53. | Liberia | 2011 | 500 | AFRO | **0.4** | 1977 | NA | — |
| 54. | Madagascar | 2016 | 450 | AFRO | **3.1** | 1992-1993 | NA | — |
| 55. | Malaysia | 2019 | 10750 | WPRO | **0.8** | 2017 | NA | — |
| 56. | Maldives | 2017 | 9400 | SEARO | **2.1** | 1984 | NA | — |
| 57. | Mali | 2005 | 520 | AFRO | **2.2** | 1983 | NA | — |
| 58. | Marshall Islands | 1989 | 2170 | WPRO | NA | — | NA | — |
| 59. | Mauritania | 2014 | 1780 | AFRO | **2.0** | 1990 | NA | — |
| 60. | Mauritius | 2013 | 10090 | AFRO | **4.9** | 1993 | NA | — |
| 61. | Mexico | 2014 | 10950 | AMRO / PAHO | **1.97** | 2010 | 2.6 | 2018 |
| 62. | Monaco | 2012 | 115700 | EURO | NA | — | NA | — |
| 63. | Montenegro | 2001 | 1710 | EURO | NA | — | 3.4 | 2006 |
| 64. | Morocco | 2019 | 3510 | EMRO | **4.82** | 2012 | NA | — |
| 65. | Mozambique | 2017 | 480 | AFRO | **0.78** | 2015 | NA | — |
| 66. | Nauru | 2007 | 3010 | WPRO | NA | — | NA | — |
| 67. | Nepal | 2002 | 230 | SEARO | **1.2** | 1994 | 0.5 | 2004 |
| 68. | Netherlands | 1992 | 23040 | EURO | **0.9** | 1992 | 0.8 | 2002 |
| 69. | New Caledonia | 2017 | 35550 | WPRO | **2.08** | 2012 | NA | — |
| 70. | Nicaragua | 2019 | 1930 | AMRO / PAHO | **1.5** | 2002 | NA | — |
| 71. | Niger | 2015 | 560 | AFRO | **1.3** | 1997 | NA | — |
| 72. | Nigeria | 2021 | 2110 | AFRO | **1.76** | 2012-2013 | NA | — |
| 73. | Niue | 1969 | 8820 | WPRO | NA | — | 1.8 | 1995 |
| 74. | Northern Mariana Islands | 1995 | 8820 | WPRO | NA | — | NA | — |
| 75. | Norway | 1981 | 1610 | EURO | **4.5** | 1979 | 0.8 | 2022 |
| 76. | Oman | 2019 | 17810 | EMRO | **1.3** | 2006 | NA | — |
| 77. | Pakistan | 2005 | 830 | EMRO | **0.9** | 1991 | 0.97 | 2014 |
| 78. | Palau | 2003 | 7740 | WPRO | NA | — | NA | — |
| 79. | Panama | 1995 | 3430 | AMRO / PAHO | **4.2** | 1989 | 3.6 | 2008 |
| 80. | Paraguay | 1992 | 1490 | AMRO / PAHO | **5.9** | 1983 | 2.8 | 2008 |
| 81. | Peru | 1999 | 2030 | AMRO / PAHO | **7.0** | 1990 | 3.7 | 2002 |
| 82. | Philippines | 2018 | 3610 | WPRO | **2.54** | 2018 | NA | — |
| 83. | Poland | 2021 | 17470 | EURO | **2.35** | 2019 | NA | — |
| 84. | Portugal | 2017 | 23180 | EURO | **1.18** | 2013-2014 | NA | — |
| 85. | Qatar | 2019 | 65700 | EMRO | **1.03** | 2011 | NA | — |
| 86. | Romania | 2023 | 16660 | EURO | **2.1** | 2008-2009 | NA | — |
| 87. | Rwanda | 2019 | 720 | AFRO | **2.09** | 2016 | NA | — |
| 88. | Sao Tome and Principe | 1976 | 1440 | AFRO | NA | — | 1.628 | 2021 |
| 89. | Saudi Arabia | 2017 | 25550 | EMRO | **2.82** | 2014 | 1.93 | 2022 |
| 90. | Senegal | 2001 | 1430 | AFRO | **1.2** | 1994 | 0.6 | 2011 |
| 91. | Seychelles | 2019 | 17620 | AFRO | **1.5** | 2005 | NA | — |
| 92. | South Africa | 2018 | 6350 | AFRO | **1.12** | 2017 | NA | — |
| 93. | Spain | 2021 | 30110 | EURO | **0.58** | 2020 | NA | — |
| 94. | Sri Lanka | 2018 | 4350 | SEARO | **0.6** | 2015-2016 | NA | — |
| 95. | St. Helena | 2014 | 2270 | AFRO | NA | — | NA | — |
| 96. | St. Kitts and Nevis | 2010 | 15970 | AMRO / PAHO | **2.6** | 1998 | NA | — |
| 97. | St. Vincent and the Grenadines | 2007 | 6030 | AMRO / PAHO | **3.25** | 1991 | NA | — |
| 98. | Sudan | 2009 | 1310 | EMRO | **0.5** | 2007-2008 | NA | — |
| 99. | Suriname | 2006 | 5730 | AMRO / PAHO | **1.9** | 2002 | NA | — |
| 100. | Tajikistan | 2018 | 1000 | EURO | **1.2** | 1985-1990 | NA | — |
| 101. | Tanzania | 2018 | 960 | AFRO | **0.24** | 2007 | 0.4 | 2020 |
| 102. | Thailand | 2017 | 5800 | SEARO | **1.4** | 2017 | NA | — |
| 103. | Timor-Leste | 2023 | 2020 | SEARO | NA | — | NA | — |
| 104. | Togo | 2019 | 830 | AFRO | **0.5** | 1999 | NA | — |
| 105. | Tonga | 2013 | 4330 | WPRO | **3.1** | 1998 | NA | — |
| 106. | Tunisia | 2018 | 2150 | EMRO | **1.3** | 1994 | NA | — |
| 107. | Türkiye | 2002 | 9550 | EURO | **1.5** | 1996 | 1.9 | 2004-2005 |
| 108. | Tuvalu | 2009 | 4870 | WPRO | **2.4** | 1986 | NA | — |
| 109. | United Arab Emirates | 2017 | 15824 | EMRO | **1.8** | 2013 | NA | — |
| 110. | Uganda | 2014 | 760 | AFRO | **0.9** | 2002 | NA | — |
| 111. | UK | 2018 | 42130 | EURO | **0.8** | 2017 | NA | — |
| 112. | Uruguay | 1990 | 5210 | AMRO / PAHO | NA | — | 1.23 | 2010-2011 |
| 113. | Vanuatu | 2015 | 2980 | WPRO | **1.2** | 1991 | NA | — |
| 114. | Wallis and Futuna | 2017 | -11674 | WPRO | NA | — | 1.6 | 2023 |
| 115. | Zambia | 2018 | 1390 | AFRO | **2.3** | 1982 | NA | — |
| 116. | Zimbabwe | 2022 | 1960 | AFRO | **0.66-1.29** | NR (pub. 2013) | NA | — |
| 117. | Western Samoa | 1998 | 8820 | WPRO | **2.5** | 1994 | NA | — |

SSBT, sugar-sweetened beverage tax; GNI, gross national income; WHO, World Health Organization; DMFT, mean number of decayed, missing because of caries, and filled permanent teeth; NA, no eligible value identified in the sources searched; NR, survey year not reported. The data-availability categories indicate whether an eligible age-12 DMFT value was identified before the SSBT only (Before), after the SSBT only (After), at both time points (Both), or at neither time point (Neither). Values from the same calendar year as SSBT implementation were treated as pre-/at-implementation because exact survey and policy dates were not consistently available. Post-SSBT values represent the most recent eligible age-12 estimate identified after implementation in the sources searched.

# Appendix Table 2. SSBT characteristics in countries implementing policy.

|  | Country | Oral Health Reference | Products | SSBT Policy Objective Articulation | Health Reasons | Economic Reasons |
| --- | --- | --- | --- | --- | --- | --- |
| 1. | American Samoa | Explicit | All soft drinks or non-alcoholic carbonated beverages [1] | Health and Economic reasons [2] | Reducing dental disease, unhealthy weight gain, type 2 diabetes, metabolic syndrome, gout, etc. | Financing for health |
| 2. | Argentina | Oral Health not Addressed | Non-alcoholic beverages, carbonated or not; fruit and vegetable juices<100%; syrups, extracts, and concentrates for soft drinks [3] | Health reasons [4] | Reducing NCD, obesity and diabetes | - |
| 3. | Azerbaijan | Oral Health not Addressed | Energy drinks [5] | Health reasons [6] | Reducing NCD, obesity and diabetes | - |
| 4. | Bahrain | Oral Health not Addressed | Any aerated beverage (except unflavoured aerated water), concentrates, powder, gel, or extracts for preparing aerated beverage [7] | Health reasons [8] | Reducing NCD, obesity and diabetes | - |
| 5. | Bangladesh | Oral Health not Addressed | Mineral and aerated waters, juices, concentrates [9] | Health reasons [10] | Reducing NCD, obesity and diabetes | - |
| 6. | Barbados | Oral Health not Addressed | Mineral and aerated waters, including waters, fruit juices and drinks, flavoured milk, malt beverages, beverages containing cocoa; flavoured or coloured sugar syrups [11] | Health reasons [12] | Reducing NCD, obesity and diabetes | - |
| 7. | Belgium | Oral Health not Addressed | Mineral and aerated waters, (exception unflavoured soy drinks), substances in intended for the manufacture of non-alcoholic drinks [13] | Health reasons [14] | Reducing NCD, obesity and diabetes | - |
| 8. | Belize | Oral Health not Addressed | Mineral and aerated waters [15] | Health reasons [16] | Reducing NCD, obesity and diabetes | - |
| 9. | Benin | Oral Health not Addressed | Any non-alcoholic beverage [*17*] | Health reasons [18] | Reducing NCD, obesity and diabetes | - |
| 10. | Bermuda | Oral Health not Addressed | Mineral and aerated waters containing added sugar; Preparations for making beverages containing added sugar; added sugar syrups; candies, pure sugar, syrups, chocolate, and added sugar cocoa [19] | Health reasons [20] | Reducing NCD, obesity and diabetes | - |
| 11. | Bolivia | Oral Health not Addressed | Mineral and aerated waters containing added sugar or other sweeteners or flavoured [21] | Health reasons [22] | Reducing NCD, obesity and diabetes | - |
| 12. | Brazil | Oral Health not Addressed | Waters (whether sweetened or not), carbonates, sports drinks, energy drinks [23] | Health reasons [24] | reducing NCD, obesity and diabetes | - |
| 13. | Brunei Darussalam | Explicit | All sugar-sweetened soft drinks [25] | Health and Economic reasons [26] | Reducing obesity, diabetes, other NCDs, and oral health problems. | financing for health |
| 14. | Burkina Faso | Oral Health not Addressed | Non-alcoholic beverages (excluding fruit and vegetable juices produced in Burkina Faso) [27] | Health reasons [28] | Reducing NCD, obesity and diabetes |  |
| 15. | Burundi | Oral Health not Addressed | Lemonades, sodas, fruit juices, and other non-alcoholic beverages [27] | Health reasons [29] | Reducing NCD, obesity and diabetes |  |
| 16. | Cabo Verde | Oral Health not Addressed | Mineral and aerated waters, containing added sugar or other sweetening matter, or flavoured [27] | Health reasons [30] | Reducing NCD, obesity and diabetes |  |
| 17. | Cambodia | Explicit | All types of beverages, including soft drinks [31] | Health and: Economic reasons [32] | Reducing obesity, diabetes, other non-communicable diseases, and oral health problems. | financing for health |
| 18. | Cameroon | Oral Health not Addressed | Domestic carbonated beverages; imported all non-alcoholic beverages [33] | Health reasonс [34] | Reducing NCD, obesity and diabetes |  |
| 19. | Central African Republic | Oral Health not Addressed | Imported non-alcoholic beverages, excluding mineral water [35] | Health reasons [36] | Reducing NCD, obesity and diabetes |  |
| 20. | Chad | Oral Health not Addressed | Sweetened or not beverages and carbonated waters [37] | Health reasons [38] | Reducing NCD, obesity and diabetes |  |
| 21. | Chile | Oral Health not Addressed | All non-alcoholic drinks with added sweeteners including energy drinks and waters [39] | Health reasons [40] | Reducing NCD, obesity and diabetes |  |
| 22. | Congo, Rep. | Oral Health not Addressed | Fruit and vegetable juices, and drinks based on fruit or vegetable juices; lemonades; other sweetened drinks, flavoured or not; natural and aerated mineral water [41] | Health reasons [42] | Reducing NCD, obesity and diabetes |  |
| 23. | Cook island | Explicit | Beverages containing added sugars [43] | Health and Economic reason [44] | Reducing dental disease, unhealthy weight gain, type 2 diabetes, metabolic syndrome, gout, etc | financing for health |
| 24. | Costa Rica | Oral Health not Addressed | All packaged beverages without alcohol content except milk [45] | Health reasons [46] | Reducing NCD, obesity and diabetes |  |
| 25. | Côte d'Ivoire | Oral Health not Addressed | Energy drinks and other non-alcoholic beverages, excluding water [47] | Health reasons [48] | Reducing NCD, obesity and diabetes |  |
| 26. | Croatia | Oral Health not Addressed | Non-alcoholic beverages, syrups, powders and granules; fruit juices, fruit base, herbal extracts, cereals or whey, artificial non-alcoholic beverages and low-energy non-alcoholic beverage [49] | Health reasons [50] | Reducing NCD, obesity and diabetes |  |
| 27. | Dominica | Oral Health not Addressed | Soft drinks and other sweetened drinks, including energy drinks, beverages containing cocoa, malt beverages, and foods with high sugar content [51] | Health reasons [52] | Reducing NCD, obesity and diabetes |  |
| 28. | Ecuador | Oral Health not Addressed | Non-alcoholic beverages and sodas, including syrups and concentrates [53] | Health reasons [54] | Reducing NCD, obesity and diabetes |  |
| 29. | Egypt | Oral Health not Addressed | Non-alcoholic beverages [55] | Health reasons [56] | Reducing NCD, obesity and diabetes |  |
| 30. | El Salvador | Oral Health not Addressed | All carbonates, all other SSBs except those containing >20% milk [57] | Health reasons [58] | Reducing NCD, obesity and diabetes |  |
| 31. | Eritrea | Oral Health not Addressed | Mineral water, lemonade, flavoured carbonated waters, syrup [59] | Health reasons [60] | Reducing NCD, obesity and diabetes |  |
| 32. | Estonia | Oral Health not Addressed | carbonated soft drinks with added sugar or other sweeteners, juices, sports and energy drinks, and milk-based sweetened drinks [61] | Health reasons [62] | Reducing NCD, obesity and diabetes |  |
| 33. | Ethiopia | Oral Health not Addressed | All types of waters, and soft drink powders [63] | Health reasons [64] | Reducing NCD, obesity and diabetes |  |
| 34. | Fiji | Explicit | Carbonated and noncarbonated drinks sweetened with sugar or artificial sweeteners; (excluded milk-based drinks); imported fruit juices [65] | Health and Economic reasons [66] | Reducing dental disease, unhealthy weight gain, type 2 diabetes, metabolic syndrome, gout, etc. | financing for health |
| 35. | Finland | Oral Health not Addressed | Water beverages, juices, concentrates and powders for preparing beverages, sports drinks, energy gels, and soy and oat beverages [67] | Health reasons [68] | Reducing NCD, obesity and diabetes |  |
| 36. | France | Oral Health not Addressed | Non-alcoholic beverages with added sugar or sweeteners including sodas, fruit drinks, flavoured waters, and ‘light’ drinks [69] | Health reasons [70] | Reducing NCD, obesity and diabetes |  |
| 37. | French Polynesia | Explicit | All sweetened drinks [71] | Health and Economic reason [72] | Reducing dental disease, unhealthy weight gain, type 2 diabetes, metabolic syndrome, gout, etc | financing for health |
| 38. | Gabon | Oral Health not Addressed | Sweetened drinks [27] | Health reasons [73] | Reducing NCD, obesity and diabetes |  |
| 39. | Gambia, The | Oral Health not Addressed | Fruit juices and other non-alcoholic beverages [74] | Health reasons [75] | Reducing NCD, obesity and diabetes |  |
| 40. | Ghana | Oral Health not Addressed | Waters with/without added sugar, sweeteners, and other non-alcoholic beverages; Fruit juices [76] | Health reasons [77] | Reducing NCD, obesity and diabetes |  |
| 41. | Grenada | Oral Health not Addressed | Sweetened beverages [78] | Health reasons [79] | Reducing NCD, obesity and diabetes |  |
| 42. | Guatemala | Oral Health not Addressed | Carbonated drinks, isotonic or sports drinks, juices and nectars, yogurts, concentrated or powdered preparations for the production of beverages and bottled natural water [80] | Health reasons [81] | Reducing NCD, obesity and diabetes |  |
| 43. | Guinea-Bissau | Oral Health not Addressed | Beverages, mineral, aerated or flavoured waters. Sweetened milks, milk or yoghurt drinks [27] | Health reasons [82] | Reducing NCD, obesity and diabetes |  |
| 44. | Honduras | Explicit | Soft drinks, fruit-based sugar-added drinks [83] | Health reasons [84] | Reducing NCD, obesity and diabetes |  |
| 45. | Hungary | Oral Health not Addressed | Soft drinks ≥ 8g sugar/100ml; excl. fruit and vegetable juices and nectars with at least 25% fruit or vegetable content, and products made using at least 50% milk-based material; concentrates used to sweeten drinks [85] | Health reasons [86] | Reducing NCD, obesity and diabetes |  |
| 46. | India | Oral Health not Addressed | Carbonated waters containing added sugar or other sweeteners, including carbonated SSB, lemonades, caffeinated beverages, and carbonated juice-based drinks [87] | Health reasons [88] | Reducing NCD, obesity and diabetes |  |
| 47. | Ireland | Secondary | Non-alcoholic, water-based, and fruit-based drinks with added sugar content ≥5 g/100 ml; Plant protein drinks and drinks containing milk fats with <119mg calcium/100ml [27] | Health and Economic reasons [89] | Reducing NCD, obesity and diabetes | financing for health |
| 48. | Isle of Man | Secondary | Any prepackaged soft drink with added sugar containing at least 5 g/100 ml [27] | Health and Economic reasons [90] | Reducing NCD, obesity diabetes | financing for health |
| 49. | Kenya | Oral Health not Addressed | All soft drinks [27] | Health reasons [91] | Reducing NCD, obesity diabetes |  |
| 50. | Kiribati | Explicit | Non-alcoholic beverages, mineral and aerated waters that contain added sugar, other sweeteners, or flavourings [27] | Health and  Economic reasons [92] | Reducing dental disease, unhealthy weight gain, type 2 diabetes, metabolic syndrome, gout, etc. | financing for health |
| 51. | Lao PDR | Explicit | Carbonated drinks, soda, mineral water, fruit juice and other non-alcoholic beverages, energy drinks [93] | Health and Economic reasons [94] | Reducing obesity, diabetes, other NCDs, and oral health problems | financing for health |
| 52. | Latvia | Oral Health not Addressed | Drinks with added sugar, sweetener, or other flavouring [95] | Health reasons [96] | Reducing NCD, obesity diabetes |  |
| 53. | Liberia | Oral Health not Addressed | imported carbonates, energy drinks, juices, low calorie sweened beverages and waters [97] | Health reasons [98] | Reducing NCD, obesity and diabetes |  |
| 54. | Madagascar | Oral Health not Addressed | Fruit and vegetable juices and drinks, carbonates, energy drinks, juices, low calorie sweened beverages, carbonated unsweetened waters [99] | Health reasons [100] | Reducing NCD, obesity and diabetes |  |
| 55. | Malaysia | Explicit | Carbonated, flavoured, and other non-alcoholic drinks, milk-based drinks, fruit and vegetable juices, and chocolate-, soy-, malt-, tea-, coffee-, and ginseng- based premixes [27] | Health and Economic reasons [101] | Reducing obesity, diabetes, other NCDs, and oral health problems | financing for health |
| 56. | Maldives | Secondary | Energy drinks, carbonates, low calorie sweetened beverages, and unsweetened waters [102] | Health reasons [103] | Reducing NCD, obesity and diabetes |  |
| 57. | Mali | Oral Health not Addressed | Carbonated waters, flavoured or containing added sugar or other sweetening matter, juices, syrups and concentrates [104] | Health reasons [105] | Reducing NCD, obesity and diabetes |  |
| 58. | Marshall Islands | Explicit | SSB, including sweetened juices, concentrates, and low-calorie sweetened beverages [27] | Health and Economic reason | Reducing dental disease, weight gain, type 2 diabetes, metabolic syndrome, gout, etc. | financing for health |
| 59. | Mauritania | Oral Health not Addressed | Imported mineral waters, sweetened dairy and yoghurt products [27] | Health reasons [106] | Reducing NCD, obesity and diabetes |  |
| 60. | Mauritius | Oral Health not Addressed | All SSBs, imported or locally produced, including juices, milk-based beverages, and soft drinks [107] | Health reasons [108] | Reducing NCD, obesity and diabetes |  |
| 61. | Mexico | Oral Health not Addressed | Non-alcoholic beverages with added sugar [27] | Health reasons [109] | Reducing NCD, obesity and diabetes |  |
| 62. | Monaco | Oral Health not Addressed | Non-alcoholic beverages including beverages containing synthetic sweeteners or added sugars, and sparkling and still waters [110] | Health reasons [111] | Reducing NCD, obesity and diabetes |  |
| 63. | Montenegro | Oral Health not Addressed | Carbonated water with added sugar or other sweeteners or aromatisation agents [112] | Health reasons [113] | Reducing NCD, obesity and diabetes |  |
| 64. | Morocco | Oral Health not Addressed | Lemonades, flavoured or unflavoured carbonated or non-carbonated water, mineral water, and table water [27] | Health reasons [114] | Reducing NCD, obesity and diabetes |  |
| 65. | Mozambique | Oral Health not Addressed | Sweetened waters [115] | Health reasons [116] | Reducing NCD, obesity and diabetes |  |
| 66. | Nauru | Explicit | Carbonated and non-carbonated soft drinks and fruit drinks containing added sugar (not sugar free or zero sugar soft drinks), milk and dairy products containing added sugar (but not soy milk or natural milk dairy products) [117] | Health and Economic reason | Reducing dental disease, weight gain, type 2 diabetes, metabolic syndrome, gout, etc. | financing for health |
| 67. | Nepal | Oral Health not Addressed | Carbonates, concentrates, energy drinks, juices, milk drinks, low calorie sweetened beverages and waters [118] | Health reasons [119] | Reducing NCD, obesity and diabetes |  |
| 68. | Netherlands | Oral Health not Addressed | Non-alcoholic beverages, fruit and vegetable juices, mineral water, lemonade, syrups, iced teas, whey drinks, non-alcoholic liquors [120] | Health reasons [121] | Reducing NCD, obesity and diabetes |  |
| 69. | New Caledonia | Explicit | All SSB, excluding soy milk [122] | Health and Economic reason | Reducing dental disease, weight gain, type 2 diabetes, metabolic syndrome, gout, etc. | financing for health |
| 70. | Nicaragua | Oral Health not Addressed | All non-alcoholic beverages, sweetened or not-sweetened, carbonated or not [123] | Health reasons [124] | Reducing NCD, obesity and diabetes |  |
| 71. | Niger | Oral Health not Addressed | Non-alcoholic beverages, fruit juice, extracts, essences or concentrates of tea and coffee [125] | Health reasons [126] | Reducing NCD, obesity and diabetes |  |
| 72. | Nigeria | Oral Health not Addressed | Non-alcoholic, carbonated and sugar-sweetened beverages, diluted concentrates and soft drinks [27] | Health reasons [127] | Reducing NCD, obesity and diabetes |  |
| 73. | Niue | Explicit | Sweetened beverages excluding flavoured water and flavoured milk [128] | Health and Economic reason | Reducing dental disease, weight gain, type 2 diabetes, metabolic syndrome, gout, etc. | financing for health |
| 74. | Northern Mariana Islands | Explicit | Soft drinks, carbonated or non-carbonated non-alcoholic beverages [129] | Health and Economic reason | Reducing dental disease, weight gain, type 2 diabetes, metabolic syndrome, gout, etc. | financing for health |
| 75. | Norway | Oral Health not Addressed | Mineral, aerated or flavoured waters with added sugars or non-sugar sweetener [130] | Health reasons [131,132] | Reducing NCD, obesity and diabetes |  |
| 76. | Oman | Oral Health not Addressed | Juices, sports drinks, fruit/malt syrups, pre-mixed ready to serve coffee and tea drinks, concentrates, powders, gels, extracts [27] | Health reasons [133] | Reducing NCD, obesity and diabetes |  |
| 77. | Pakistan | Oral Health not Addressed | Waters with/without added sugar or artificial sweeteners excluding mineral water; flavoured milk and yoghurt; sugary fruit juices, syrups and squashes [134] | Health reasons [135] | Reducing NCD, obesity and diabetes |  |
| 78. | Palau | Explicit | Carbonated soft drinks, bottled water [136] | Health and Economic reason [137] | Reducing dental disease, weight gain, type 2 diabetes, metabolic syndrome, gout, etc. | financing for health |
| 79. | Panama | Oral Health not Addressed | Non-alcoholic beverages containing added sugar or sweeteners and concentrated syrups [138] | Health reasons [139] | Reducing NCD, obesity and diabetes |  |
| 80. | Paraguay | Oral Health not Addressed | juice of fruits, soda and beverages up to 2% alcohol [27] | Health reasons [140] | Reducing NCD, obesity and diabetes |  |
| 81. | Peru | Oral Health not Addressed | Non-alcoholic beverages, sweetened waters, and 0% alcohol beer [27] | Health reasons [141] | Reducing NCD, obesity and diabetes |  |
| 82. | Philippines | Explicit | Beverages made with caloric and non-caloric sweeteners, sweetened juice drinks, sweetened tea, carbonates, flavoured water, energy and sports drinks, powdered drinks, juice, tea or coffee, cereal and grain beverages [27] | Health and Economic reason [142] | Reducing dental disease, weight gain, type 2 diabetes, metabolic syndrome, gout, etc. | financing for health |
| 83. | Poland | Oral Health not Addressed | Mineral, aerated or flavoured waters with added sugars, and non-sugar sweetener, energy drinks [27] | Health reasons [143] | Reducing NCD, obesity and diabetes |  |
| 84. | Portugal | Oral Health not Addressed | Mineral, flavoured, and aerated waters containing added sugar or sweeteners, concentrates [27] | Health reasons [144] | Reducing NCD, obesity and diabetes |  |
| 85. | Qatar | Oral Health not Addressed | Aerated beverage (except unflavoured aerated water): carbonated water with added sugar, other sweeteners or flavours, concentrates, powder, gel or extracts to make beverages [27] | Health reasons [145] | Reducing NCD, obesity and diabetes |  |
| 86. | Romania | Oral Health not Addressed | All non-alcoholic beverages with added sugars [27] | Health reasons [146] | Reducing NCD, obesity and diabetes |  |
| 87. | Rwanda | Oral Health not Addressed | Lemonade, soda, juices [27] | Health reasons [147] | Reducing NCD, obesity and diabetes |  |
| 88. | Sao Tome and Principe | Oral Health not Addressed | Carbonates, concentrates, energy drinks, juices, milk drinks and low-calorie sweetened beverages [148] | Health reasons [149] | Reducing NCD, obesity and diabetes |  |
| 89. | Saudi Arabia | Oral Health not Addressed | All drinks with added sugars [27] | Health reasons [150] | Reducing NCD, obesity and diabetes |  |
| 90. | Senegal | Oral Health not Addressed | carbonated water; lemonades, sodas and other carbonated beverages; fruit and vegetable juices [27] | Health reasons [151] | Reducing NCD, obesity and diabetes |  |
| 91. | Seychelles | Oral Health not Addressed | Beverages containing >5 g sugar/100 ml, including milk drinks [27] | Health reasons [152] | Reducing NCD, obesity and diabetes |  |
| 92. | South Africa | Oral Health not Addressed | Sugary beverages>4g/100ml [153] | Health reasons [154, 155] | Reducing NCD, obesity and diabetes |  |
| 93. | Spain | Oral Health not Addressed | soft drinks, flavoured water, chocolate drinks, sports drinks, cold tea and coffee drinks, energy drinks, fruit nectar drinks, vegetable drinks, sweetened milk and milk alternatives, milkshakes, and milk drinks with fruit juice [27] | Health reasons [156] | Reducing NCD, obesity and diabetes |  |
| 94. | Sri Lanka | Secondary | All SSBs, excluded milk-based drinks [27] | Health reasons [157] | Reducing NCD, obesity and diabetes |  |
| 95. | St. Helena | Oral Health not Addressed | Water, sugar-free yoghurt [27] | Health reasons [158] | Reducing NCD, obesity and diabetes |  |
| 96. | St. Kitts and Nevis | Oral Health not Addressed | Mineral and aerated waters, containing added sugar or other sweetener, or flavour, non-alcoholic beverages, beverages containing cocoa and malt beverages [27] | Health reasons [159] | Reducing NCD, obesity and diabetes |  |
| 97. | St. Vincent and the Grenadines | Oral Health not Addressed | Mineral and aerated waters, containing added sugar or other sweetener or flavour, and other non-alcoholic beverages [160] | Health reasons [161] | Reducing NCD, obesity and diabetes |  |
| 98. | Sudan | Oral Health not Addressed | Fruit juices, natural or artificial mineral waters, soft drinks and other flavoured waters [27] | Health reasons [162] | Reducing NCD, obesity and diabetes |  |
| 99. | Suriname | Oral Health not Addressed | Non-alcoholic beverages [27] | Health reasons [163] | Reducing NCD, obesity and diabetes |  |
| 100. | Tajikistan | Oral Health not Addressed | Carbonates, concentrates, energy drinks, low calorie sweetened beverages, waters [164] | Health reasons [165] | Reducing NCD, obesity and diabetes |  |
| 101. | Tanzania | Oral Health not Addressed | Non-alcoholic beverages with/without added sugar or sweeteners [27] | Health reasons [166] | Reducing NCD, obesity and diabetes |  |
| 102. | Thailand | Explicit | Non-alcoholic beverages, juices, coffee and tea drinks, concentrates for preparation, whether or not containing added sugar or sweeteners [27] | Health and Economic reason [167] | Reducing dental disease, weight gain, type 2 diabetes, metabolic syndrome, gout, etc. | financing for health |
| 103. | Timor-Leste | Oral Health not Addressed | Beverages, mineral aerated or flavoured water [27] | Health reasons [168] | Reducing NCD, obesity and diabetes |  |
| 104. | Togo | Oral Health not Addressed | Sweetened non-alcoholic drinks; fresh fruit juice [27] | Health reasons [169] | Reducing NCD, obesity and diabetes |  |
| 105. | Tonga | Explicit | Full sugar and artificially sweetened soft drinks, energy drinks [170] | Health and Economic reason [171] | Reducing dental disease, weight gain, type 2 diabetes, metabolic syndrome, gout, etc. | financing for health |
| 106. | Tunisia | Oral Health not Addressed | Mineral and aerated waters containing added sugar, sweeteners, flavours; non-alcoholic drinks, fruit and vegetable juices unfermented without addition of alcohol, preparations containing cocoa, extracts, essences and concentrates of coffee, tea or mate [27] | Health reasons [172] | Reducing NCD, obesity and diabetes |  |
| 107. | Türkiye | Oral Health not Addressed | Cola sodas; fruit juices, nectars and drinks excl. 100% juice; lemonades; energy drinks; sports drinks; cold tea and coffee; tonic [27] | Health reasons [173] | Reducing NCD, obesity and diabetes |  |
| 108. | Tuvalu | Explicit | Carbonates, energy drinks, low calorie sweetened beverages [27] | Health and Economic reason [174] | Reducing dental disease, weight gain, type 2 diabetes, metabolic syndrome, gout, etc. | financing for health |
| 109. | United Arab Emirates | Oral Health not Addressed | Beverages with added sugars or sweetener, including concentrates, powders for making beverage [27] | Health reasons [175] | Reducing NCD, obesity and diabetes |  |
| 110. | Uganda | Oral Health not Addressed | Non-alcoholic beverages, excluding juice made from at least 30% of pulp from fruit and vegetables grown in Uganda [176] | Health reasons [177] | Reducing NCD, obesity and diabetes |  |
| 111. | UK | Secondary | Any prepackaged soft drink with added sugar containing at least 5g of total sugars/100ml. [178] | Health and Economic reasons [179] | Reducing NCD, obesity and diabetes, children and families weight management, breastfeeding  initiatives and the promotion of oral hygiene | financing for health |
| 112. | Uruguay | Oral Health not Addressed | Carbonated and non-carbonated soft drinks, sweetened with caloric or non-caloric sweeteners, mineral waters, juices and fruit drinks, energy drinks, concentrates and syrups [180] | Health reasons [181] | Reducing NCD, obesity and diabetes |  |
| 113. | Vanuatu | Explicit | Mineral and aerated waters, containing added sugar or other sweetening matter or flavoured, and other non-alcoholic beverages [27] | Health and Economic reasons [182] | Reducing NCD, obesity and diabetes, children and families weight management, breastfeeding  initiatives and the promotion of oral hygiene | financing for health |
| 114. | Wallis and Futuna | Explicit | Carbonates, concentrates, energy drinks, juices, milk drinks, low calorie sweetened beverages, waters [183] | Health and Economic reasons | Reducing NCD, obesity and diabetes, children and families weight management, breastfeeding  initiatives and the promotion of oral hygiene | financing for health |
| 115. | Zambia | Oral Health not Addressed | Non-alcoholic beverages [27] | Health reasons [184] | Reducing NCD, obesity and diabetes |  |
| 116. | Zimbabwe | Oral Health not Addressed | Beverage, energy drink [27] | Health reasons [185] | Reducing NCD, obesity and diabetes |  |
| 117. | Western Samoa | Oral Health not Addressed | Mineral and aerated waters, containing added sugar [27] | Health reasons | Reducing NCD, obesity and diabetes |  |

**References**

1.	American Samoa Bar Association. Chapter 10 Excise Tax on Imports. 2020 [cited 2025 December 16]. https://new.asbar.org/code-annotated/11-1002-amount-of-tax-on-certain-items/.

2.	Secretariat of the Pacific Community. Sugar-sweetened beverage tax in Pacific Island countries and territories: A discussion paper. (accessed on 15 Dec 2025). https://www.spc.int/DigitalLibrary/Doc/PHD/NCD/Diabetes/Diabetes_for_Health_Professionals/Sugar_sweetened_beverage_tax_in_Pacific_Island_countries_and_territories_a_discussion_paper.pdf

3.	Ministry of Justice Republic of Argentina. InfoLEG Ley 24.674. 1996 [cited 2025 December 20]. https://servicios.infoleg.gob.ar/infolegInternet/anexos/35000-39999/38621/texact.htm .

4.	Alcaraz A, Perelli L, Rodriguez MB, Palacios A, Bardach A, Gittens-Baynes KA, et al. What does our region need in order to strengthen public policies on sugar-sweetened beverages? decision-makers’ dialogue. Rev Peru Med Exp Salud Publica. 2023;40(1):86–93.

5.	State Tax Service Under the Ministry of Economy of the Republic of Azerbaijan. 2025. [cited 2025 December 20]. https://www.taxes.gov.az/az/page/qanunlar.

6.	Global report on the use of sugar-sweetened beverage taxes, 2023. Geneva: World Health Organization; 2023. [accessed on 1 Dec 2025]. chrome-extension://efaidnbmnnnibpcajpcglclefindmkaj/https://iris.who.int/server/api/core/bitstreams/9dc39e4a-e2ba-4ae3-b10e-579101605264/content.

7.	Kingdom of Bahrain. Ministry of Interior Customs Affairs. [accessed on 1 Dec 2025]. https://www.bahraincustoms.gov.bh/ar/laws-and-regulations

8.	The Global database on the Implementation of Food and Nutrition Action (GIFNA). Bahrain Law No. (40) of 2017 Concerning Selective Tax [accessed on 1 Dec 2025]. https://gifna.who.int/countries/BHR/policies/82190 .

9.	National Board of Revenue Bangladesh. VAT Acts. 2011-2026. [accessed on 1 Dec 2025]. <https://nbr.gov.bd/regulations/acts/vat-acts/eng>

10.	The Global database on the Implementation of Food and Nutrition Action (GIFNA). Bangladesh Value Added Tax and Supplementary Duty Act, 2012 (Act No. 48 of 2012) (accessed on 1 Dec 2025). https://gifna.who.int/countries/BGD/policies/82189

11.	Presentation of the financial statement and budgetary proposals 2015 delivered by the Minister of Finance and Economic Affairs to the house of assembly Barbados. (accessed on 1 Dec 2025). <https://www.barbadosparliament.com/uploads/document/d1efb84aac6a7abe4c6c0efcf8ceedd2.pdf>

12.	The Global database on the Implementation of Food and Nutrition Action (GIFNA). Barbados Revenue Authority. Excise Tax on Sweetened Beverages 2022 (accessed on 1 Dec 2025). https://gifna.who.int/countries/BRB/policies/83691

13.	Federal Public Service Finance Belgium. Customs and Excise. Excise Duty Rates. (accessed on 1 Dec 2025). https://finances.belgium.be/fr/douanes_accises/entreprises/accises/informations-generales/taux-droits-daccise

14.	The Global database on the Implementation of Food and Nutrition Action (GIFNA). Belgium Law relating to the excise regime for non-alcoholic beverages and coffee. (accessed on 1 Dec 2025). https://gifna.who.int/countries/BEL/policies/26240

15.	Customs and Excise Act Chapter 48 Belize. 2000. (accessed on 1 Dec 2025). https://www.customs.gov.bz/download/customsexcise_duties.pdf

16.	The Global database on the Implementation of Food and Nutrition Action (GIFNA). Belize Customs and Excise Duties Act 2000 (accessed on 1 Dec 2025). https://gifna.who.int/countries/BLZ/policies/82191

17.	Republic of Benin. National Assembly Report (27^th^ December 2011). (accessed on 1 Dec 2025). <https://sgg.gouv.bj/doc/loi-2011-43/>

18.	The Global database on the Implementation of Food and Nutrition Action (GIFNA). Ministry of Finance and Economic Affairs. General Tax Code of Benin 2021 (accessed on 1 Dec 2025).

[https://gifna.who.int/countries/BEN/policies/132519](https://gifna.who.int/countries/BEN/policies/132519 19)

[19](https://gifna.who.int/countries/BEN/policies/132519 19).	Government of Bermuda. The Sugar Tax 2018. (accessed on 1 Dec 2025). <https://www.gov.bm/articles/sugar-tax#:~:text=The%20amendments%20introduced%20today%20propose,from%201st%20April%202019>.

20.	Government of Bermuda. Ministry of Health. Sugar Tax Consultation Final Report. (accessed on 1 Dec 2025). <https://www.gov.bm/sites/default/files/Sugar%20Tax%20Final%20Report%20and%20Consultation%20Document%20for%20Website%20160629_0.pdf>

21.	National Tax Service Bolivia. Compendium of Bolivian Tax Regulations. Bolivian Taxes. 2025 (accessed on 1 Dec 2025). https://www.impuestos.gob.bo/

22.	The Global database on the Implementation of Food and Nutrition Action (GIFNA). National Tax Service Bolivia. 2017 (accessed on 1 Dec 2025). <https://gifna.who.int/countries/BOL/policies/25413>

23.	The Chamber of Deputies Brazil. 1967. (accessed on 1 Dec 2025). <https://www2.camara.leg.br/legin/fed/declei/1960-1969/decreto-lei-116-a-27-janeiro-1967-375797-publicacaooriginal-1-pe.html>

24.	The Global database on the Implementation of Food and Nutrition Action (GIFNA). The Tax Incidence Table of Industrialized Products Brazil. (accessed on 1 Dec 2025). <https://gifna.who.int/countries/BRA/policies/132043>

25.	Brunei Darussalam Government Gazette. 2006, 2017 (accessed on 1 Dec 2025). <https://www.agc.gov.bn/AGC%20Images/LAWS/Gazette_PDF/2017/EN/S027.pdf>

26.	The Global database on the Implementation of Food and Nutrition Action (GIFNA). Royal Customs and Excise Department, Ministry of Finance Brunei Darussalam. Tariff and Trade Classification. 2017 (accessed on 1 Dec 2025). https://gifna.who.int/countries/BRN/policies/82193

27.	World Bank Group. Global SSB Tax Database. 2025. (accessed September 2025). https://datacatalog.worldbank.org/search/dataset/0063310/global-ssb-tax-database

28.	The Global database on the Implementation of Food and Nutrition Action (GIFNA). Government of Burkina Fasso. Law No. 058-2017/AN establishing the General Tax Code of Burkina Faso 2017. (accessed on 1 Dec 2025). https://gifna.who.int/countries/BFA/policies/126208

29.	The Global database on the Implementation of Food and Nutrition Action (GIFNA). Republic of Burundi, Office of the President. Law No. 1/36 establishing the general budget of the Republic of Burundi for the 2015 fiscal year 2014. (accessed on 1 Dec 2025). https://gifna.who.int/countries/BDI/policies/130091

30.	Cabo Verde Boletim Official. Supplement. National Assembly Law 44/IX/2018. 2018 (accessed on 1 Dec 2025). <https://www.mf.gov.cv/documents/92878/0/bo_31-12-2018_89.pdf/ac195b04-bba2-1d30-447a-b0216a33aea9?t=1554290555785>

31.	Ministry of Economy and Finance of the Kingdom of Cambodia. 2020. Prakas No. 012 MEF.Prk (accessed on 1 Dec 2025). <https://www.tax.gov.kh/u6rhf7ogbi6/gdtstream/423b1fac-111a-4297-947a-cb251d46eeb3>

32.	The Global database on the Implementation of Food and Nutrition Action (GIFNA). The General Department of Taxation Cambodia. Notification 28271 on the adjustment of the special tax rate on non-alcoholic beverage products of domestic enterprises. 2023 (accessed on 1 Dec 2025). https://gifna.who.int/countries/KHM/policies/126220

33.	Directorate General of Taxation Cameroon 2019 (accessed on 1 Dec 2025). <https://www.impots.cm/uploads/Telechargement/GENERAL_TAX_CODE_DGI_2019.pdf>

34.	The Global database on the Implementation of Food and Nutrition Action (GIFNA). Directorate General of Taxation Cameroon 2022 (accessed on 1 Dec 2025). https://gifna.who.int/countries/CMR/policies/126209

35.	Ministry of Finances Central African Republic 2025. (accessed on 1 Dec 2025). <http://finances.gouv.cf/finances/les-lois-de-finances-ldf?order=field_date_creation&sort=desc>

36.	The Global database on the Implementation of Food and Nutrition Action (GIFNA). Ministray of Financies Budget Government of Central African Repiblic 2018 (accessed on 1 Dec 2025). https://gifna.who.int/countries/CAF/policies/126210

37.	Ministry of Financies, Budget and Economy Republic of Chad (accessed on 1 Dec 2025). <https://finances.gouv.td/index.php/publications/lois-des-finances>

38.	The Global database on the Implementation of Food and Nutrition Action (GIFNA). Presidency of the Republic of Chad Law No. 037/PR/2018 Concerning the General State Budget for 2019 (accessed on 1 Dec 2025). https://gifna.who.int/countries/TCD/policies/38182

39.	Library of the National Congress of Chile Decree 1637 2014. (accessed on 1 Dec 2025). <https://www.bcn.cl/leychile/navegar?idNorma=1071503>

40.	The Global database on the Implementation of Food and Nutrition Action (GIFNA). Law 20780. Tax reform 2014 (accessed on 1 Dec 2025). https://gifna.who.int/countries/CHL/policies/26237

41.	Republic of Congo Consitution. Law No. 18-002 enacting the Excise Code 2018 (accessed on 1 Dec 2025). <https://www.leganet.cd/Legislation/Dfiscal/Accisices/Loi%2013%20mars%202018.pdf>

42.	The Global database on the Implementation of Food and Nutrition Action (GIFNA). Finance Law Republic of Congo. Law No. 34 2014. (accessed on 1 Dec 2025). https://gifna.who.int/countries/COG/policies/128145

43.	The Working Tariff Document of the Cook Islands. 2018. (accessed on 1 Dec 2025). <https://cook-islands.tradeportal.org/media/HS%202017%20Consolidated%20-%201st%20June%202018.pdf>

44.	The Global database on the Implementation of Food and Nutrition Action (GIFNA). Parliament of the Cook Islands Customs Revenue and Border Protection Act 2012 (accessed on 1 Dec 2025). https://gifna.who.int/countries/COK/policies/26291

45.	Attorney General's Office of the Republic of Costa Rica. (accessed on 1 Dec 2025). <http://www.pgrweb.go.cr/>

46.	The Global database on the Implementation of Food and Nutrition Action (GIFNA). Ministry of Finance and Presidential Offcie Costa Rica Update of Specific Taxes 2019 (accessed on 1 Dec 2025). https://gifna.who.int/countries/CRI/policies/82196

47.	Ministry of Finances and Budget Côte d'Ivoire. General Tax Code 2025 (accessed on 1 Dec 2025). <http://dgi.cgici.com/indexs.htm>

48.	The Global database on the Implementation of Food and Nutrition Action (GIFNA). Government of Côte d'Ivoire. Finance Act for 2018. (accessed on 1 Dec 2025). https://gifna.who.int/countries/CIV/policies/82194

49.	Official Gazzette of the Republic of Croatia. Tax on coffee and soft drinks 2020. (accessed on 1 Dec 2025). <https://narodne-novine.nn.hr/clanci/sluzbeni/2020_02_22_532.html>

50.	The Global database on the Implementation of Food and Nutrition Action (GIFNA). Law on Special Tax on Coffee and Soft Drinks & Ordinance on Special Tax on Coffee and Non-Alcoholic Beverages 2013 (accessed on 1 Dec 2025). https://gifna.who.int/countries/HRV/policies/82203

51.	Commonwealth of Dominica. Excise Tax 2015. (accessed on 1 Dec 2025). <http://www.dominica.gov.dm/laws/2015/Excise%20Tax%20%20(Amd)%20Order,%202015.pdf>

52.	The Global database on the Implementation of Food and Nutrition Action (GIFNA). House of Assembly Dominica Excise Tax ORDER NO. 28 OF 2015. (accessed on 1 Dec 2025). https://gifna.who.int/countries/DMA/policies/26239

53.	National Legal Directorate Republic of Equador, Department of Tax Regulations. 2016. (accessed on 1 Dec 2025). <http://www.asamblea.go.cr/sd/Documents/CEDIL/ALERTA%20MAYO%202018/LOEFP%C3%BAblicasEcuador.pdf>

54.	The Global database on the Implementation of Food and Nutrition Action (GIFNA). National Legal Directorate, Department of Tax Regulations Internal Tax Regime 2014. (accessed on 1 Dec 2025) https://gifna.who.int/countries/ECU/policies/25416

55.	Ministry of Finance Arab Republic of Egypt. (accessed on 1 Dec 2025)	<https://mof.gov.eg/ar/posts/regulations/5fe868ec7bfc600007cbeeb7/%D9%82%D8%A7%D9%86%D9%88%D9%86%20%D8%A7%D9%84%D9%82%D9%8A%D9%85%D8%A9%20%D8%A7%D9%84%D9%85%D8%B6%D8%A7%D9%81%D8%A9>

56.	The Global database on the Implementation of Food and Nutrition Action (GIFNA). Ministry of Finance Egypt Law No. 67 of 2016 promulgating the Value Added Tax Law 2016. (accessed on 1 Dec 2025) https://gifna.who.int/countries/EGY/policies/126221

57.	Department of Judicial Documentation. Supreme Court of Justice of El Salvador. Updated 2025 (accessed on 1 Dec 2025). <https://www.jurisprudencia.gob.sv/DocumentosBoveda/D/2/2000-2009/2009/12/888FD.PDF>

58.	Global Nutrition Report 2022. Country Nutrition Profile El Salvador (accessed on 1 Dec 2025). https://globalnutritionreport.org/resources/nutrition-profiles/latin-america-and-caribbean/central-america/el-salvador/

59.	Gazette of Eritrean Laws. Government of Eritrea. Proclamation to Amend the Income Tax Eritrea 2001 (accessed on 1 Dec 2025). <https://assets.tobaccocontrollaws.org/uploads/legislation/Eritrea/Eritrea-Tax-Amdt.-Proc.-national.pdf>

60.	The Global database on the Implementation of Food and Nutrition Action (GIFNA). Gazette of Eritrean Laws: Proclamations and Legal Notices. Proclamation No. 64/1994. Sales and Excise Tax Proclamation (accessed on 1 Dec 2025) <https://gifna.who.int/countries/ERI/policies/126231>

61.	European Comission. Health Promotion and Disease Prevention Knowledge Gateway. (accessed on 1 Dec 2025) <https://knowledge4policy.ec.europa.eu/health-promotion-knowledge-gateway/sugars-sweeteners-10_en?utm_source>

62.	The Global database on the Implementation of Food and Nutrition Action (GIFNA). Policies in Estonia. (accessed on 1 Dec 2025). <https://gifna.who.int/countries/EST/policies>

63.	Ethiopian Legal Information Portal. Law Ethiopia. (accessed on 1 Dec 2025). <https://www.lawethiopia.com/images/EXCISE%20TAX%20PROCLAMATION.pdf>

64.	The Global database on the Implementation of Food and Nutrition Action (GIFNA). Ministry of Finance Government of Ethiopia Excise Tax Proclamation No. 1186/2020 (accessed on 1 Dec 2025). <https://gifna.who.int/countries/ETH/policies/40429>

65.	Fiji Revenue and Customs Service. Excise Act 1986 (accessed on 1 Dec 2025). <https://www.frcs.org.fj/wp-content/uploads/2020/08/Excise-Act-1986-Revised-31-July-2020.pdf>

66.	The Global database on the Implementation of Food and Nutrition Action (GIFNA). Parlament of Republic of Fiji. Excise Act 2020 (accessed on 1 Dec 2025). <https://gifna.who.int/countries/FJI/policies/130106>

67.	Finlex. Ministry of Justice Finland. Soft Drinks Tax Act. 2010. (accessed on 1 Dec 2025). <https://finlex.fi/fi/laki/ajantasa/2010/20101127>

68.	The Global database on the Implementation of Food and Nutrition Action (GIFNA). Treasury of Finland. Act amending the Law on excise duty on confectionery, ice cream and soft drinks, Policy - 1535/2016 (accessed on 1 Dec 2025). <https://gifna.who.int/countries/FIN/policies/26224>

69.	French Republic. Legifrance. General Tax Code. 2012 (accessed on 1 Dec 2025). <https://www.legifrance.gouv.fr/codes/article_lc/LEGIARTI000025051331/2011-12-30>

70.	The Global database on the Implementation of Food and Nutrition Action (GIFNA). National Assemby, the Presidential Offcie. General Tax Code 2018 (accessed on 1 Dec 2025). <https://gifna.who.int/countries/FRA/policies/26225>

71.	Directorate of Taxes and Public Contributions of French Polynesia. Tax Code (accessed on 1 Dec 2025). <https://www.impot-polynesie.gov.pf/code/2-section-ii-base-dimposition-et-taux>

72.	The Global database on the Implementation of Food and Nutrition Action (GIFNA). Ministry of Solidarity and Health French Polynesia 2018 (accessed on 1 Dec 2025). <https://gifna.who.int/countries/PYF/policies/39753>

73.	The Global database on the Implementation of Food and Nutrition Action (GIFNA). Official Journal of the Republic of Gabon Law No. 17.022 establishing the State budget 2018 (accessed on 1 Dec 2025). <https://gifna.who.int/countries/GAB/policies/126211>

74.	Trade Policies and Practicies by Measure. Trade Policy Review Gambia. 2010. (accessed on 1 Dec 2025). <https://docs.wto.org/dol2fe/Pages/SS/directdoc.aspx?filename=Q:/WT/TPR/S233-04.pdf&Open=True>

75.	The Global database on the Implementation of Food and Nutrition Action (GIFNA). Government of Gambia. he Customs and Excise Act, 2010 (accessed on 1 Dec 2025). <https://gifna.who.int/countries/GMB/policies/127668>

76.	Ghana Revenue Authority. Excise Duty. 2014 (accessed on 1 Dec 2025). <https://gra.gov.gh/domestic-tax/tax-types/excise-duty/>

77.	The Global database on the Implementation of Food and Nutrition Action (GIFNA). Government of Ghana Excise Duty Act 2014 (Act 878) (accessed on 1 Dec 2025). <https://gifna.who.int/countries/GHA/policies/126233>

78.	Ministry of Finance Grenada. Sweetened Beverages Tax. 2023 (accessed on 1 Dec 2025). <https://www.finance.gd/Sweetened%20Beverages%20Tax%20Fact%20Sheet_March2023.pdf?utm_source>

79.	Government of Grenada, Ministry of Finance. Sweetened Beverages Tax Fact Sheet, March 2023. St. George’s: Ministry of Finance, Grenada. (accessed on 1 Dec 2025). <https://www.finance.gd/Sweetened%20Beverages%20Tax%20Fact%20Sheet_March2023.pdf?utm_source>

80.	The Congress of The Republic of Guatemala. Tax and Taxable Acts 2002 (accessed on 1 Dec 2025). <https://docs.bvsalud.org/leisref/2018/03/170/gtm-decreto-09-2002-impuesto-selectivo-bebidas-gaseosas.pdf#:~:text=Se%20aprueba%20el%20impuesto%20espec%C3%ADfico,nacional%20o%20que%20sean%20importadas>

81.	Chacon V, Paraje G, Barnoya J, Chaloupka FJ. Own-price, cross-price, and expenditure elasticities on sugar-sweetened beverages in Guatemala. PLoS One. 2018 Oct 22;13(10):e0205931. doi: 10.1371/journal.pone.0205931

82.	General Directorate of Contributions and Taxes Guinea Bissao. 2021 (accessed on 1 Dec 2025). <https://gifna.who.int/countries/GNB/policies/126215>

83.	Pan American Health Organization, World Health Organization, World Bank Group. Sugar-sweetened Beverages and Prepackaged Foods: the Impact of Taxation on Price, Consumption, and Revenues and its Contribution to Achieving the Sustainable Development Goals in Central America, Panama, and the Dominican Republic. 2020 (accessed on 1 Dec 2025). <https://thedocs.worldbank.org/en/doc/611961599658512658-0090022020/original/TF0A4082FullreportSugarSweetDrinksEnglishFinal20201.pdf?utm_source>

84.	The Global database on the Implementation of Food and Nutrition Action (GIFNA). La Gaceta Honduras. Decree 17-2010 Law on strengthening income, social equity and rationalization of public spending. 2010 (accessed on 1 Dec 2025). <https://gifna.who.int/countries/HND/policies/82201>

85.	National Legislation Hungary 2011 (accessed on 1 Dec 2025). <http://njt.hu/cgi_bin/njt_doc.cgi?docid=139165.357212#foot1>

86.	World Health Organization. Fiscal Policies for Diet and Prevention of Noncommunicable Diseases. 2015 (accessed on 1 Dec 2025). Available from : <https://www.who.int/docs/default-source/obesity/fiscal-policies-for-diet-and-the-prevention-of-noncommunicable-diseases-0.pdf?sfvrsn=84ee20c_2>

87.	Ministry of Finance Department of Revenue India. (accessed on 1 Dec 2025). <https://cbic-gst.gov.in/gst-goods-services-rates.html>

88.	The Global database on the Implementation of Food and Nutrition Action (GIFNA). The Gazette of India. Central Excise Tariff 2016-17 (accessed on 1 Dec 2025). <https://gifna.who.int/countries/IND/policies/26280>

89.	The Global database on the Implementation of Food and Nutrition Action (GIFNA). The Irish Statue Book Finance Act 2017 (accessed on 1 Dec 2025). <https://gifna.who.int/countries/IRL/policies/82205>

90.	UK Public General Acts Finance Act 2017 Isle of Man: import and export of chargeable soft drinks. (accessed on 1 Dec 2025). <https://www.legislation.gov.uk/ukpga/2017/10/section/58A>

91.	Kenya Gazette Supplement. Acts, the Excise Duty Act 2015. (accessed on 1 Dec 2025). <http://kenyalaw.org/kl/fileadmin/pdfdownloads/Acts/ExciseDutyActNo23of2015.pdf>

92.	The Global database on the Implementation of Food and Nutrition Action (GIFNA). Ministry of Finance and Economic Development Government of Kiribati. Excise Tax Rates Order 2014 (accessed on 1 Dec 2025). <https://gifna.who.int/countries/KIR/policies/82208>

93.	Lao People's Democratic Republic. Tax Law 2011 (accessed on 1 Dec 2025). <https://www.wto.org/english/thewto_e/acc_e/lao_e/wtacclao36a1_leg_2.pdf>

94.	The Global database on the Implementation of Food and Nutrition Action (GIFNA). Ministry of Finance Lao PDR Law on Excise Taxes 2020 (accessed on 1 Dec 2025). <https://gifna.who.int/countries/LAO/policies/126241>

95	The Republic of Latvia. Amendments to the Law on Excise Duty.2019. (accessed on 1 Dec 2025). <https://titania.saeima.lv/LIVS13/saeimalivs13.nsf/webSasaiste?OpenView&restricttocategory=266/Lp13>

96.	The Global database on the Implementation of Food and Nutrition Action (GIFNA). Law on excise duties Latvia 2003 (accessed on 1 Dec 2025) <https://gifna.who.int/countries/LVA/policies/129352>

97.	Liberia Revenue Code as Amended. 2020. (accessed on 1 Dec 2025) <https://revenue.lra.gov.lr/wp-content/uploads/2021/08/REVENUE-CODE-LIBERIA-REVENUE-CODE-AMENDEMENT-2020-min.pdf>

98.	The Global database on the Implementation of Food and Nutrition Action (GIFNA). Ministry of Foreign Affairs Government of Liberia. Excise Tax Law 2018 (accessed on 1 Dec 2025). <https://gifna.who.int/countries/LBR/policies/38190>

99.	Republic of Madagaskar Finance Law. 2015. (accessed on 1 Dec 2025). <http://www.mef.gov.mg/assets/vendor/ckeditor/plugins/kcfinder/upload/files/Loi%20n%C2%B0%202015-050%20du%2029%20de%CC%81cembre%202015%20Portant%20Loi%20de%20Finances%20pour%202016.pdf>

100.	The Global database on the Implementation of Food and Nutrition Action (GIFNA). Republic of Madagaskar Finance Law 2022 (accessed on 1 Dec 2025). <https://gifna.who.int/countries/MDG/policies/82216>

101.	The Global database on the Implementation of Food and Nutrition Action (GIFNA). Ministry of Finance Malaysia. Excise Duties (Amendment) Order 2022 (accessed on 1 Dec 2025). <https://gifna.who.int/countries/MYS/policies/129354>

102.	Maldives Customs Service. Tariff. 2025 (accessed on 1 Dec 2025). <https://www.customs.gov.mv/EServices/Tariff>

103.	The Global database on the Implementation of Food and Nutrition Action (GIFNA). Ministry of Health. National Multi-sectoral Action Plan for the Prevention and Control of Noncommunicable Diseases in Maldives (2023-2031) (accessed on 1 Dec 2025). <https://gifna.who.int/countries/MDV/policies/136610>

104.	Official Gazette Republic of Mali. Acts of the Republic Of Mali. 2005 (accessed on 1 Dec 2025). <http://sgg-mali.ml/JO/2005/mali-jo-2005-14.pdf>

105.	The Global database on the Implementation of Food and Nutrition Action (GIFNA). Prime Minister's Office - General Secretariat of the Government of Mali. Special Tax on Certain Products 2005 (accessed on 1 Dec 2025) <https://gifna.who.int/countries/MLI/policies/25353>

106.	Global Nutrition Report. Country Nutrition Profiles Mauritania 2022. (accessed on 1 Dec 2025). Available from : <https://globalnutritionreport.org/resources/nutrition-profiles/africa/western-africa/mauritania/>]

107.	Mauritius Revenue Authority. Excise Duty on Sugar Content of Sugar Sweetened Product. 2025. (accessed on 1 Dec 2025). <https://www.mra.mu/index.php/customs1/more-topics/excise-tax-on-sugar-content-of-sugar-sweetened-non-alcoholic-beverages>

108.	The Global database on the Implementation of Food and Nutrition Action (GIFNA). Mauritius. The Finance Act 2009. (accessed on 1 Dec 2025). <https://gifna.who.int/countries/MUS/policies/26218>

109.	The Global database on the Implementation of Food and Nutrition Action (GIFNA). Secretariat Of Finance and Public Credit Mexico. Decree amending, adding to, and repealing various provisions of the Value Added Tax Law; of the Special Tax on Production and Services Law 2013 (accessed on 1 Dec 2025). Available online: <https://gifna.who.int/countries/MEX/policies/25417>

110.	Government of Monaco. Ministerial Decree No. 2018-64. 2018 (accessed on 1 Dec 2025). <https://extranet.who.int/ncdccs/Data/MCO_NCD_MCO_Arr%c3%aat%c3%a9%20Minist%c3%a9riel%20No.%202018-64%20du%2030%20janvier%202018%20fixant%20les%20tarids%20des%20droits%20d'accises.pdf>

111.	World Health Organization. Regional Office for Europe. Sugar-sweetened beverage taxes in the WHO European region. Success through lessons learned and challenges faced. (accessed on 1 Dec 2025). <https://iris.who.int/bitstream/handle/10665/351781/WHO-EURO-2022-4781-44544-63081-eng.pdf?sequence=1&isAllowed=y>

112.	Constitution of the Republic of Montenegro. Law on Excise Taxes. 2001 (accessed on 1 Dec 2025). <https://extranet.who.int/ncdccs/Data/MNE_NCD_MNE_unofficial%20translation%20Law%20on%20Excise%20Taxes%20(2017).pdf>

113.	The Global database on the Implementation of Food and Nutrition Action (GIFNA). The Parliament of Montenegro. Decree on the promulgation of the law on amendments to the law on excise duties. 2011 (accessed on 1 Dec 2025). Available online: <https://gifna.who.int/countries/MNE/policies/82217>

114.	The Global database on the Implementation of Food and Nutrition Action (GIFNA). Kingdom of Morocco. Finance Law No. 70-19. 2020. (accessed on 1 Dec 2025). Available online: (<https://gifna.who.int/countries/MAR/policies/82211>

115.	Tax Authority of Mozambique. Law No. 17. 2017. (accessed on 1 Dec 2025). <http://www.at.gov.mz/por/Media/Files/Lei-n1-17-2017-de-28-de-Dezembro>

116.	The Global database on the Implementation of Food and Nutrition Action (GIFNA). President of the Republic Mozambique. Tax Code on Specific Consumptions. 2017. (accessed on 1 Dec 2025). <https://gifna.who.int/countries/MOZ/policies/82218>

117.	Nauru’s Online Legal Database. 2025 (accessed on 1 Dec 2025). <http://ronlaw.gov.nr/nauru_lpms/index.php/act/view/964>

118.	Government of Nepal. Ministry of Finance. Inland Revenue Department. 2021 (accessed on 1 Dec 2025). <https://ird.gov.np/tax-laws/category/acts/exciseact>

119.	The Global database on the Implementation of Food and Nutrition Action (GIFNA). Government of Nepal. he Excise Duty Act 2022. (accessed on 1 Dec 2025). <https://gifna.who.int/countries/NPL/policies/82228>

120.	Ministry of Finance Netheralnds. Customs. Excise duty and consumption tax rate list. 2025. (accessed on 1 Dec 2025). <https://www.belastingdienst.nl/wps/wcm/connect/bldcontentnl/themaoverstijgend/brochures_en_publicaties/tarievenlijst_accijns_en_verbruiksbelastingen>

121.	The Global database on the Implementation of Food and Nutrition Action (GIFNA). Kingdom of the Netherland. The Act introducing an excise duty on non-alcoholic drinks 1971. (accessed on 1 Dec 2025). <https://gifna.who.int/countries/NLD/policies/126229>

122.	Government of New Caledonia. The general consumption tax in the customs clearance document. 2018. (accessed on 1 Dec 2025). <https://douane.gouv.nc/la-taxe-generale-sur-la-consommation-tgc-dans-lacte-de-dedouanement>

123.	List of Consumer Goods Taxed With ISC. Nicaragua. (accessed on 1 Dec 2025). [http://legislacion.asamblea.gob.ni/SILEG/Iniciativas.nsf/0/3636a2c1dc3dae2606257654006000c2/$FILE/ANEXO_I[1].pdf](http://legislacion.asamblea.gob.ni/SILEG/Iniciativas.nsf/0/3636a2c1dc3dae2606257654006000c2/$FILE/ANEXO_I%5b1%5d.pdf)

124.	The Global database on the Implementation of Food and Nutrition Action (GIFNA). National Assembly of the Republic of Nicaragua. Law No. 987, Law of reforms and additions to Law No. 822, Tax agreement Law. (accessed on 1 Dec 2025). [h2019.ttps://gifna.who.int/countries/NIC/policies/82224](https://gifna.who.int/countries/NIC/policies/82224)

125.	Government of Niger. Budget Plan for 2020. 2019. (accessed on 1 Dec 2025). <https://www.impots.gouv.ne/media/loi/2020.pdf>

126. The Global database on the Implementation of Food and Nutrition Action (GIFNA). Republic of Niger. Law 2015-38 amending the General Tax Code 2015 (accessed on 1 Dec 2025). <https://gifna.who.int/countries/NER/policies/26176>

127.	The Global database on the Implementation of Food and Nutrition Action (GIFNA). Federal Republic of Nigeria. Finance Act 2021 (accessed on 1 Dec 2025). <https://gifna.who.int/countries/NGA/policies/130365>

128.	Niue Customs and Tariff Regulations. 2016. (accessed on 1 Dec 2025). <https://niue.tradeportal.org/media/Reg%202016-04%20Niue%20Customs%20and%20Tarriff%20(General%20Amendment)%20Regulations%202016.PDF>

129.	Northern Mariana Islands Commonwealth Legislature. Revenue and Taxation. 1994 (accessed on 1 Dec 2025). <https://www.cnmilaw.org/pdf/public_laws/09/pl09-22.pdf>

130.	The Norwegian Tax Administration. Sugar Tax. (accessed on 1 Dec 2025). <https://www.skatteetaten.no/en/business-and-organisation/vat-and-duties/excise-duties/about-the-excise-duties/sugar/?utm>

131.	The Global database on the Implementation of Food and Nutrition Action (GIFNA). Ministry of Finance Norwegian Government. Amendment to the Regulation on Excise Duties. 2006. (accessed on 1 Dec 2025). <https://gifna.who.int/countries/NOR/policies/26178>

132.	Royal Ministry of Finance. Norwegian excise duties on chocolate and sugar products and non-alcoholic beverages. 2018. (accessed on 1 Dec 2025). <https://www.stortinget.no/globalassets/pdf/dokumentserien/2017-2018/dok15-201718-2140-vedlegg5.pdf>

133.	The Global database on the Implementation of Food and Nutrition Action (GIFNA). Ministry of Finance Oman. Oman Ministerial decree No. 112/2019 On determining the value and types of selective goods and the category of tax levied on each. 2019 (accessed on 1 Dec 2025). <https://gifna.who.int/countries/OMN/policies/38194>

134.	Federal Board of Revenue Government of Pakistan. Finance Act 2023. (accessed on 1 Dec 2025). <https://www.fbr.gov.pk/finance-supplementary-act/173791>

135.	The Global database on the Implementation of Food and Nutrition Action (GIFNA). Parliament of Pakistan. Federal Excise Act. 2005 (accessed on 1 Dec 2025). <https://gifna.who.int/countries/PAK/policies/82230>

136.	National Treasury of the Republic of Palau. Revenue and Taxation. (accessed on 1 Dec 2025). <https://www.palaugov.pw/wp-content/uploads/2022/04/Palau-National-Code-Title-40.pdf>

137.	The Global database on the Implementation of Food and Nutrition Action (GIFNA). Republic of Palau Public Law. Excise Tax Act 2021. (accessed on 1 Dec 2025).  <https://gifna.who.int/countries/PLW/policies/136782>

138.	Republic Of Panama Legislative Assembly. 1995. (accessed on 1 Dec 2025). <https://docs.panama.justia.com/federales/leyes/45-de-1995-nov-15-1995.pdf>

139.	The Global database on the Implementation of Food and Nutrition Action (GIFNA). Republic of Panama, Law No 45. 1995.(accessed on 1 Dec 2025). <https://gifna.who.int/countries/PAN/policies/82231>

140.	The Global database on the Implementation of Food and Nutrition Action (GIFNA). Treasury Government of Paraguay. Law No. 125/91 1991. (accessed on 1 Dec 2025). Available online: <https://gifna.who.int/countries/PRY/policies/82234>

141.	The Global database on the Implementation of Food and Nutrition Action (GIFNA) Ministry of Economy and Finance Government of Peru. Supreme Decree No. 091-2018-EF. 2018. (accessed on 1 Dec 2025). Available online: <https://gifna.who.int/countries/PER/policies/82232>)

142.	The Global database on the Implementation of Food and Nutrition Action (GIFNA) Philipines ax Reform for Acceleration and Inclusion. 2017. (accessed on 1 Dec 2025). Available online: <https://gifna.who.int/countries/PHL/policies/82233>

143.	The Global database on the Implementation of Food and Nutrition Action (GIFNA) Poland Amending certain acts in connection with the promotion of health-promoting choices of consumer 2020. (accessed on 1 Dec 2025). Available online: https://gifna.who.int/countries/POL/policies/42604

144.	The Global database on the Implementation of Food and Nutrition Action (GIFNA) Portugal. ection III Amendment to the Code on Excise Tax 2016 (accessed on 1 Dec 2025). https://gifna.who.int/countries/PRT/policies/26242

145.	The Global database on the Implementation of Food and Nutrition Action (GIFNA). Qatar General Tax Authority. Law No. (25) of 2018 Concerning Excise Tax (accessed on 1 Dec 2025). Available online: https://gifna.who.int/countries/QAT/policies/82236

146.	The Global database on the Implementation of Food and Nutrition Action (GIFNA). Romania. Law no. 296 2023 (accessed on 1 Dec 2025) Available online: <https://gifna.who.int/countries/ROU/policies/129101>

147.	The Global database on the Implementation of Food and Nutrition Action (GIFNA). Rwanda Government  Law N° 26/2006 of 27/05/2006 determining and establishing consumption tax on some imported and locally manufactured products 2006 (accessed on 1 Dec 2025). Available online: <https://gifna.who.int/countries/RWA/policies/82237>

148.	National Assembley Sao Tome and Principe 2017. <https://www2.camara.leg.br/saotomeeprincipe/diarios-da-an/x-legislatura/ii-serie/6.a-sessao-legislativa/DAN32-IIS.pdf/view>

149.	The Global database on the Implementation of Food and Nutrition Action (GIFNA). Government of the Democratic Republic of Sao Tome and Principe. Decree-Law No. 20/76 Consumption Tax Rates 1976. (accessed on 1 Dec 2025) <https://gifna.who.int/countries/STP/policies/38186> ()

150.	The Global database on the Implementation of Food and Nutrition Action (GIFNA). Saudi Arabia Zakat, Tax and Customs Authority. Implementing regulations for the excise tax system 2019. (accessed on 1 Dec 2025). <https://gifna.who.int/countries/SAU/policies/82239>

151.	The Global database on the Implementation of Food and Nutrition Action (GIFNA). Senegal Law No. 2018-10 General Tax Code 2018 (accessed on 1 Dec 2025) <https://gifna.who.int/countries/SEN/policies/82242>

152.	The Global database on the Implementation of Food and Nutrition Action (GIFNA). Seychelles Excise Tax (Imposition of Sugar Tax on Drinks) Regulations, 2019. (accessed on 1 Dec 2025). https://gifna.who.int/countries/SYC/policies/129348

153.	South African Revenue Service. Health Promotion Levy on Sugary Beverages 2025. (accessed on 1 Dec 2025). <https://www.sars.gov.za/customs-and-excise/excise/health-promotion-levy-on-sugary-beverages/>

154.	Stacey N, Edoka I, Hofman K, Swart EC, Popkin B, Ng SW. Changes in beverage purchases following the announcement and implementation of South Africa's Health Promotion Levy: an observational study. Lancet Planet Health. 2021 Apr;5(4):e200-e208. doi: 10.1016/S2542-5196(20)30304-1

155.	Kadungure A, Loewenson R (2023) Taxing for health: taxes on sugar-sweetened beverages in east and southern African countries, EQUINET discussion paper 130, TARSC, EQUINET, Harare. Available online: https://equinetafrica.org/sites/default/files/uploads/documents/EQ%20Disss%20130%20SSB%20taxation%20July2023_0.pdf (accessed on 1 Dec 2025)

156.	The Global database on the Implementation of Food and Nutrition Action (GIFNA). Department of the Vice Presidency and of Economy and Finance Spain. Law 5/2017 (accessed on 1 Dec 2025). https://gifna.who.int/countries/ESP/policies/26271

157.	The Global database on the Implementation of Food and Nutrition Action (GIFNA). Sri Lanka Excise (Special Provisions) Act, No. 13 Of 1989. 2017. (accessed on 1 Dec 2025). <https://gifna.who.int/countries/LKA/policies/82210>

158.	Sant Helena Government. 2018. (accessed on 1 Dec 2025) https://www.sainthelena.gov.sh/2018/public-announcements/changes-to-customs-excise-duty/?utm

159.	The Global database on the Implementation of Food and Nutrition Action (GIFNA). Saint Kitts and Nevis Excise Tax Act, 2010. (accessed on 1 Dec 2025). https://gifna.who.int/countries/KNA/policies/82209

160.	St. Vincent and the Grenadines. Excise Tax Act 2007, (accessed on 1 Dec 2025). <https://customs.gov.vc/downloads/act-excise-tax-ch430.pdf>

161.	The Global database on the Implementation of Food and Nutrition Action (GIFNA). Government of Saint Vincent and the Grenadines. Excise Tax Act 2009. (accessed on 1 Dec 2025). https://gifna.who.int/countries/VCT/policies/38191

162.	Training and Research Support Centre (TARSC) in the Regional Network for Equity in Health in East and Southern Africa (EQUINET). July 2023. "Taxing for health: taxes on sugar-sweetened beverages in east and southern African countries". Available online: https://equinetafrica.org/sites/default/files/uploads/documents/EQ%20Disss%20130%20SSB%20taxation%20July2023_0.pdf (accessed on 1 Dec 2025).

163.	The Global database on the Implementation of Food and Nutrition Action (GIFNA). Suriname Non-alcoholic Beverage Excise Tax Act. 2006. (accessed on 1 Dec 2025). <https://gifna.who.int/countries/SUR/policies/82245>

164.	The Government of the Republic of Tajikistan. Decision on Excise Tax Rates 2018 (accessed on 1 Dec 2025). <https://andoz.tj/docs/postanovleniya-pravitelstvo/Resolution__%E2%84%96302_gov_RT_tj.pdf>

165.	The Global database on the Implementation of Food and Nutrition Action (GIFNA). Approval of excise tax rates for some goods produced in and imported into the Republic of Tajikistan 2014. (accessed on 1 Dec 2025). https://gifna.who.int/countries/TJK/policies/82247.

166.	The Global database on the Implementation of Food and Nutrition Action (GIFNA). United Republic of Tanzania. The Finance Act 2018. (accessed on 1 Dec 2025). https://gifna.who.int/countries/TZA/policies/38178

167.	The Global database on the Implementation of Food and Nutrition Action (GIFNA). Thailand Ministerial Regulations Prescribing Excise Tariffs 2017 (accessed on 1 Dec 2025). <https://gifna.who.int/countries/THA/policies/82246>

168.	The Global database on the Implementation of Food and Nutrition Action (GIFNA). Timor-Leste. Law No. 15/2022 of December 21st General State Budget for 2023. (accessed on 1 Dec 2025). https://gifna.who.int/countries/TLS/policies/130092

169.	The Global database on the Implementation of Food and Nutrition Action (GIFNA). Togo Law No. 2018-024 establishing the General Tax Code 2018. (accessed on 1 Dec 2025). <https://gifna.who.int/countries/TGO/policies/38183>

170.	Tonga Excise Tax Order 2018. (accessed on 1 Dec 2025). <https://ago.gov.to/cms/images/LEGISLATION/SUBORDINATE/2018/2018-0017/ExciseTaxAmendmentOrder2018_1.pdf>

171.	The Global database on the Implementation of Food and Nutrition Action (GIFNA). Tonga Excise Tax (Amendment) Order 2020 (accessed on 1 Dec 2025). https://gifna.who.int/countries/TON/policies/130356

172.	The Global database on the Implementation of Food and Nutrition Action (GIFNA). Tunisia Law No 2015-53. 2015. (accessed on 1 Dec 2025). <https://gifna.who.int/countries/TUN/policies/82249>

173.	The Global database on the Implementation of Food and Nutrition Action (GIFNA). Türkiye Special Consumption Tax 2014 (accessed on 1 Dec 2025). https://gifna.who.int/countries/TUR/policies/82251

174.	The Global database on the Implementation of Food and Nutrition Action (GIFNA). Tuvalu. Customs Excise Duties Order 2020 (accessed on 1 Dec 2025). https://gifna.who.int/countries/TUV/policies/82252

175.	The Global database on the Implementation of Food and Nutrition Action (GIFNA). United Arab Emirates. Cabinet Decision No. (38) on Excise Goods, Excise Tax Rates and the Method of Calculating the Excise Price. 2017. (accessed on 1 Dec 2025). https://gifna.who.int/countries/ARE/policies/26241

176.	Uganda the Excise Duty Act 2018. (accessed on 1 Dec 2025) <https://extranet.who.int/ncdccs/Data/UGA_A3iii_s21_UGA%202018%20Excise%20Duty%20Amendment%20Act.pdf>

177.	The Global database on the Implementation of Food and Nutrition Action (GIFNA). Uganda the Excise Duty Act 2020. (accessed on 1 Dec 2025). https://gifna.who.int/countries/UGA/policies

178.	UK Statutory Instruments. The Soft Drinks Industry Levy Regulations 2018 (accessed on 1 Dec 2025). <https://www.legislation.gov.uk/uksi/2018/41/contents/made>

179.	The Global database on the Implementation of Food and Nutrition Action (GIFNA). Government of the United Kingdom. Soft Drinks Industry Levy (Finance Act 2017). (accessed on 1 Dec 2025). https://gifna.who.int/countries/GBR/policies/82199

180.	Official Information Center. Uruguayan regulations and notices. Decree No. 96/990. 1990 (accessed on 1 Dec 2025). <https://www.impo.com.uy/bases/decretos/96-1990>

181.	The Global database on the Implementation of Food and Nutrition Action (GIFNA). Uruguay. Law No. 17,151 Modifies the articles that are determined of Decree-Law No. 14,416 1999. (accessed on 1 Dec 2025). https://gifna.who.int/countries/URY/policies/82254

182.	The Global database on the Implementation of Food and Nutrition Action (GIFNA). President and Parliament of the Republic of Vanuatu. Excise (Amendment) Act No. 32. 2014. (accessed on 1 Dec 2025). https://gifna.who.int/countries/VUT/policies/36000

183.	Wallis and Fortuna. Customs Tariff. 2020. (accessed on 1 Dec 2025). <https://www.douane.wf/wp-content/uploads/2020/04/TARIF-DES-DOUANES-mis-%C3%A0-jour-le-23.04.2020-1.pdf>

184.	The Global database on the Implementation of Food and Nutrition Action (GIFNA). Government of Zambia. Customs and Excise (Amendment). 2018. (accessed on 1 Dec 2025). Available online: https://gifna.who.int/countries/ZMB/policies/38189

185.	The Global database on the Implementation of Food and Nutrition Action (GIFNA). Ministry of Finance and Economic Development, Government of Zimbabwe. Customs and Excise (Tariff) Notice. 2022. (accessed on 1 Dec 2025). https://gifna.who.int/countries/ZWE/policies/126235
